# Preparation and characterization of low-cost adsorbents for the efficient removal of malachite green using response surface modeling and reusability studies

**DOI:** 10.1038/s41598-023-31391-4

**Published:** 2023-03-18

**Authors:** Mohammed Taha Moustafa

**Affiliations:** Central Laboratory for Environmental Quality Monitoring, National Water Research Center, Shubra El Kheima 1, Al Qalyubia Governorate, 6210001 Egypt

**Keywords:** Microbiology, Environmental sciences, Nanoscience and technology

## Abstract

Malachite green used in textile and dyeing industries is a common persistent pollutant in wastewater and the environment causing major hazards to human health and aquatic organisms. In this study, the response surface methodology was applied to optimize the adsorptive removal of malachite green using nano-bentonite, MgO-impregnated clay, and *Mucor* sp. composites. The nano materials and *Mucor *sp. composite were characterized by FTIR, SEM and X-ray diffractometry. According to the obtained results, nano-bentonite exhibits a maximum MG adsorption efficiency of 98.6% at 35 °C, pH 7.0, 60 min contact time, 1.0 g/L adsorbent dosage, and 50 mg/L initial MG concentration. On the other hand, the maximum efficiency for MG adsorption on MgO-impregnated clay of 97.04% is observed at pH 9.0, 60 min contact time, 0.7 g/L adsorbent dosage, and 50 mg/L initial MG concentration. The Malachite green (MG) adsorption isotherm on MgO-impregnated clay corresponded with the Freundlich isotherm, with a correlation coefficient (R^2^) of 0.982. However, the Langmuir adsorption isotherm was a superior fit for nano-bentonite (R^2^ = 0.992). The adsorption activities of nano-bentonite and MgO-impregnated clay were fitted into a pseudo-second-order kinetic model with R^2^ of 0.996 and 0.995, respectively. Additionally, despite being recycled numerous times, the adsorbent maintained its high structural stability and removal effectiveness for nano-bentonite (94.5–86%) and MgO-impregnated clay (92–83%).

## Introduction

Water pollution caused by wastewater from textile manufacturing activities is a major global concern. One of the most difficult tasks that researchers are faced with around the world in the twenty-first century is to provide the clean water necessary for industrial, house hold, and agricultural activities^1^. Textile factories are responsible for one of the major environmental pollution issues in the world, because they discharge undesirable dye effluents^2^. The textile industry consumes 100–200 L of water per kg of textiles produced, resulting in the generation of large amounts of wastewater during the dyeing process^3^. Globally, about 280,000 tons of synthetic dyes are discharged into natural streams every year from wastewater produced by a variety industries, such as leather, food, textile, paper, cosmetic, printing, and carpet manufacturers^4^. The said discharge has an adverse impact on the visual quality of water bodies, and it interferes with the lifecycles of aquatic organisms by reducing the penetration of sunlight into the water, which inhibits photosynthesis and plant growth, thereby affecting the biological activity of aquatic animals; moreover, the synthetic dyes present in water bodies also cause soil contamination^5^. Malachite green (MG) is a synthetic dye used to dye silk, cotton, leather, wool, and paper, and it is also employed as a fungicide and disinfectant in the fish farming industry, as it affords the control of fish parasites and diseases^6^. MG is a cationic triphenylmethane compound that is highly soluble in water^7^. It is also highly toxic to mammalian cells at concentrations below 0.1 g/mL^8^. MG is characterized by a complex molecular structure, high stability, non-biodegradability, and high resistance to light and oxidizing agents^7^. When it flows into the receiving stream, this dye negatively affects the lifecycles of aquatic organisms by interfering with the physiology of the pituitary liver, gills, kidneys, intestines, gonads, and gonad vegetative cells^9^. In humans, MG inhalation can cause inflammation of the respiratory tract, while its swallowing can cause inflammation of the digestive tract^10^. MG is hazardous to humans, and mutagenic; additionally, its presence affects the immunological and reproductive systems^11^. Malachite green can be converted into leucoMalachite green and carbinol, which is toxic to humans. In fish muscles, fat, and internal organs, MG has a half-life of 10 days^12^. This cationic dye is also durable in the environment, with a half-life of 12.9–50.34 days in sediment^13^. Many technologies have been used to treat textile wastewater, including physical, chemical, and advanced treatment methods, such as membrane filtration, ion exchange, electrochemical technology, coagulation, flocculation, reverse osmosis, chemical oxidation, ozonation^14^, and biological treatment for fungi and bacteria effects^15^. However, most of these technologies have various disadvantages, including low efficiency, large capital investment, high energy consumption, high cost, non-selectiveness, unsuitability for large-scale applications, and the formation of harmful secondary sludge^16^. Among the treatment strategies, adsorption is one of the most appealing and efficient methods for removing dyes from polluted water samples. This technique provides various advantages, including a simple design, recyclable adsorbents, simple operation, non toxicity, low cost, and a modest initial investment^17^. These recyclable adsorbents include activated carbon (AC)^18^, lime peel^19^, and pumice^20^. However, there are various downsides to the different adsorbents used to purify water. For example, reusing AC requires regeneration, which is costly and limits its large-scale application in wastewater treatment. In addition, some adsorbents are effective against a limited number of dyes and are difficult to separate from treated water^21^. Reference^22^ focused on the immobilization of horseradish peroxidase onto supports such as polyamide-6 electrospun fibers, which were used for the decolorization of reactive black 5 and malachite green textile dyes from solutions imitating polluted sea waters and reached over 70%. Reference^23^ presented the application of immobilization of laccase from *Trichoderma versicolor* onto various supports, such as TiO_2_–ZrO_2_–SiO_2_, to remove the azo dye reactive black 5 (RB5), the anthraquinone dye reactive blue 4 (RB4), degradation efficiencies reaching 100%, 91%, and 77%, respectively, they gained over 70% catalytic activity of immobilized laccase on TiO_2_–ZrO_2_–SiO_2_ even after five run cycles. Recently, scientists have developed an efficient and economical adsorbent material, nano-clay polymer composites, to overcome the shortcomings of traditional purification methods for textile industry wastewater and reduce their environmental threat. Currently, clay is widely used in various industries, including cosmetics, oil exploration, pharmaceuticals, food, and papermaking, because it is easily available, nontoxic, and has the potential for ion exchange for the removal of dyes from wastewater^24^. Among the clay materials studied, bentonite has received considerable attention as an adsorbent due to its low cost, renewability, large surface area, good chemical and mechanical stability, and abundance in nature^25^. Furthermore, bentonite is mostly composed of montmorillonite^26^. Raw bentonite has poor adsorption capacity for cationic dyes, so it is modified using physical and chemical treatments. However, the negatively charged surface lattice of bentonite clay may have a superior absorption capacity for cationic dyes^27^. Chemically treated modified bentonite has been used to remove cationic basic methylene blue^28^, metal ions^29^, and crystal violet^30^. Thus, this study aims to evaluate the effectiveness of modeling of the response surface methodology, which was analyzed during the experiments to optimize and assess the interactive effects of nano-bentonite, MgO-impregnated clay, and *Mucor* sp. on MG removal. Furthermore, isotherms, pseudo-first-order and pseudo-second-order models and thermodynamic parameters were determined.

## Materials and methods

### Chemicals and materials

The bentonite used in this study was obtained from CMB Co. (Egypt). Magnesium chloride dihydrate (MgCl_2_·2H_2_O) and hydrochloric acid were provided by Sigma-Aldrich Co. (Egypt).

### Analytical measurements for nano-bentonite and MgO-impregnated clay characterization

Magnesium-impregnated clay and nano-bentonite were characterized by scanning electron microscopy (SEM) (Quanta 250 FEI Company), transmission electron microscopy (TEM) with a JEOL-JEM-2100, Fourier-transform infrared (FTIR) spectroscopy analysis performed with a Bruker-VERTEX 80 V instrument ranging from 900 to 5 cm^−1^ wavenumber range, and X-Ray diffractometry (XRD) with a PANalytical X’Pert Pro(United Kingdom).

### Preparation of the dye solution

**Figure 1:**
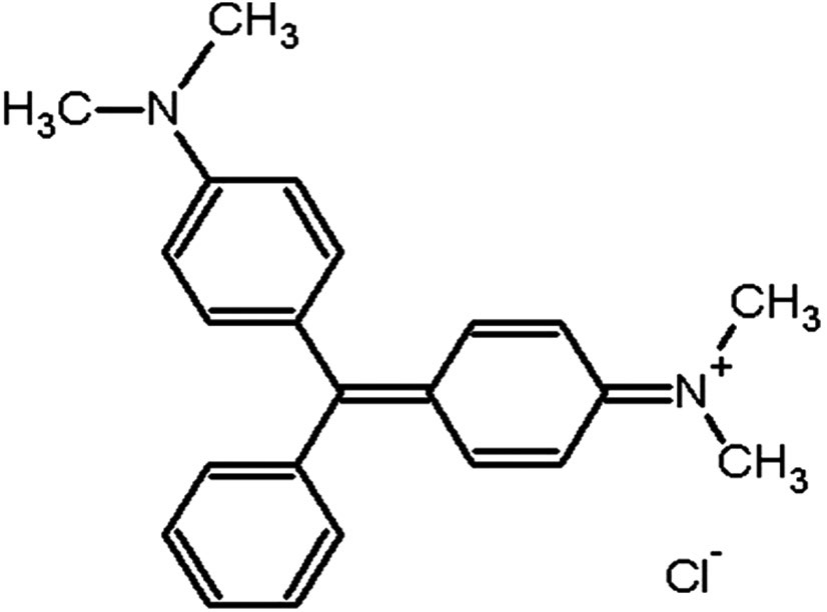
Molecular structure of Malachite green.

The cationic dye MG (Fig. 1; chemical formula: C_46_H_50_N_42_C_2_HO_4_C_2_H_2_0_4_, MW: 927.1 g/mol) was purchased from MERCK Pvt. Ltd(England). A 1 g sample of the appropriate MG was dissolved in 1000 mL of distilled water to produce an MG stock solution of 1000 mg/L concentration. The stock solution was then used to prepare MG solutions of concentrations ranging from 30 to 150 mg/L. The initial pH of the stock solution was adjusted by adding to it 0.1 M HCl or NaOH. A 50 ml aliquot of the MG stock solution was used for each of the experiments. All experiments were conducted in triplicate.

### Preparation of nano-bentonite

An amount of 21 g of bentonite powder and 100 mL of12 M HCl solution were combined, and the resulting mixture was heated in a magnetic stirrer at around 343 K and stirred at a rate of 340 rpm for 120 min. Subsequently, the obtained suspension was filtered and the precipitate was repeatedly washed with distilled water until the pH of the water used to wash the residue reached neutrality*.* The thus obtained acid-activated bentonite was dried in the oven for 5 h at a temperature of 373 K. The precipitate was then ground in a mortar to produce a powder, which was calcined in a furnace at 600 °C for 2 h^31^.

### Fabrication of MgO-impregnated clay nanocomposite

A mixture of 7 g of bentonite clay and 100 mL of 1.25 M magnesium chloride solution was stirred for 6 h. After stirring; the solution was poured into a glass petri dish and dried in an oven at 150 °C. The dried mixture was crushed to a fine powder and calcined in a muffle furnace at 450 °C for 2 h. The calcined powder was cooled, washed twice with deionized water, and dried at 70 °C for 6 h^32^.

### Determination of the zero point charge of the adsorbent

The pH point of zero surface charge characteristics of nano-bentonite and MgO-impregnated clay was determined using the following method^33^: 50 mL of 0.1 M NaCl solution was transferred into 100 mL Erlenmeyer flasks, with the initial pH (pHi) values adjusted from 3.0 to 12.0 by adding 0.1 M HCl or NaOH. Next, 0.3 g nano-bentonite and MgO-impregnated clay were added to each flask, and the suspensions were stirred continuously for 24 h. The final pH values of the supernatant liquids were assessed after 24 h. The pH _PZC_ was plotted against the difference between the initial and final pH (pHf) values. The zero point of charge (pHZPC) of the substance was considered the point where the resulting curve intersected the pHi axis at pH = 0.

### Batch adsorption experiments

Batch adsorption experiments were carried out to achieve the optimum operating conditions for removing of the MG dye. 100 mL solution of dye initial concentration was taken in 250 mL flasks and a known amount of nano-bentonite and MgO-impregnated clay, the adsorbents were added to the solutions. The mixture was shaken mechanically at a constant speed of 200 rpm using rotary shaker (Dragon LAB, skp-0330-pro, Germany).

The effects that different experimental parameters had on the efficiency of MG removal were investigated. In particular, various values were utilized for the pH (3.0, 5.0, 6.0, 7.0, 8.0, 9.0, 10.0, and 11.0), contact time (10–60 min), adsorbent dosage (0.05, 0.1, 0.2, 0.5, 0.7, and 1.0 g/L), initial dye concentration (50–250 mg/L), and temperature (298, 303, 323, and 343 K). The initial pH values were adjusted using 0.1 M HCl or 0.1 M NaOH solutions and a pH meter (Multi 9620 IDS-pH meter, WTW, Germany). Each experiment was performed three times and the averages values of the measurable were calculated and presented. Samples were taken out after the equilibrium time (60 min) and centrifuged at 4000 rpm for 25 min to completely separate the nanobentonite and MgO-impregnated clay from the solution and MG concentrations in the supernatants were determined measuring the supernatants’ absorption at the wavelength at which MG exhibits its maximum absorption (λmax = 620 nm) using a spectrophotometer (Thermo Fisher Scientific, Orion Aquamat 8000, USA). MG removal efficiency, R (%), was determined through Eq. (1):

1 $$\%R=\frac{Co-CF}{C0}\times 100,$$

where C_0_ and C_f_ represent the initial and final concentrations of the dye solution (mg/L).

The adsorption capacity (qe, mg/g) at equilibrium, was determined using Eq. (2):

2 $$qe=\frac{(Ci-C)}{M}V,$$

where Ci (mg/L) and Ce (mg/L) are the MG dye concentrations in the initial solution and at equilibrium, respectively; V (L) is the volume of the solution; and w is the mass of the adsorbent (mg).

### Equilibrium studies

In the current investigation, the equilibrium condition for the adsorption of MG on nano-bentonite and MgO-impregnated clay was described using the Langmuir, Freundlich, and Tempkin models, as given by^34^.

### Kinetic studies

Pseudo-first-order and pseudo-second-order kinetic models were utilized to analyze the kinetics of MG adsorption on the adsorbents. The pseudo-primary-order model, in its linear form is described by^35^.

### Experimental design using the response surface methodology

As a design method, the response surface methodology (RSM) is a mathematical tool that uses a second-order equation to determine the best conditions between the controllable input factors and the response variable. The effects of various factors, such as pH (X1), temperature (X2), adsorbent dosage (X3), and initial concentration (X4), on the decolorization process, were studied using the Box–Behnken design. Twenty-seven experimental runs were obtained according to the three levels of each variable; low level (− 1), level; (0) (medium) and high level (1) were used to design and analyze the experiments (Table 1). The second-order quadratic equation model was assessed to predict the optimum value between the dependent and independent factors. The correlation’s general form can be stated according to Eq. (3):

3 $${\text{Y}} = \beta_{{\text{o}}} + \mathop \sum \limits_{{{\text{i}} = 1}}^{{\text{n}}} \beta_{{\text{i}}} {\text{Xi}} + \mathop \sum \limits_{{{\text{i}} = 1}}^{{\text{n}}} \beta_{{{\text{ii}}}} {\text{Xi}}2 + \mathop \sum \limits_{{{\text{i}} - 1}}^{{{\text{n}} - 1}} \mathop \sum \limits_{{{\text{j}} - 1}}^{{\text{n}}} {\text{Bij}} {\text{Xi}} {\text{Xj}} .$$

**Table 1:** The experimental range and levels of input process variables assessed.

Variables	Code	Levels		
		− 1	0	1
pH	A	5	7	9
Dye concentration (mg/l)	B	5	50	100
Temperature (°C)	C	30	35	40
Dosage (g/l)	D	2.0	4.0	6.0

Here, Y is the predicted response factor (the removal of MG), and X is the input variable. β0, βj, βjj, and βij are the intercept, linear effect, square effect, and interaction effect, respectively. N is the quantity of input-controlling coded variable. The coefficient of determination (R^2^) and Fisher’s F-test were used to describe the quality of the quadratic model equation. Using Design-Expert 13, an analysis of variance (ANOVA) was conducted to determine the model’s statistical significance.

### Microbial toxicity

The microbial toxicity of the Malachite green dye on *Escherichia coli*, *Staphylococcus aureus*, and *Pseudomonas aeruginose* was investigated. Furthermore, using an agar well assay, the toxicity of the dye and its breakdown products were investigated. After 24 h of incubation at 37 °C, the zone of microbial growth inhibition was recorded.

### Isolation and identification of Malachite green

**Figure 2:**
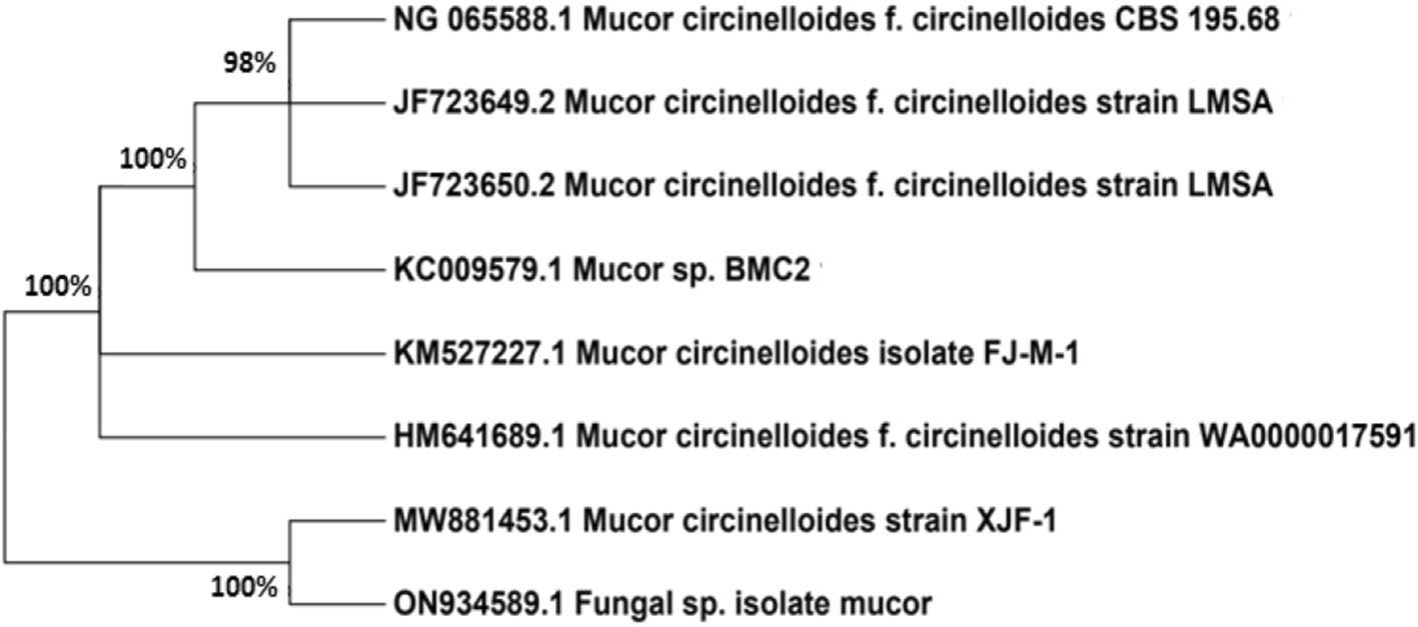
Phylogenetic tree of the fungal isolate *Mucor sp.*

A pure fungal strain was isolated from wastewater, and seven fungal strains capable of decolorizing the Malachite green dye were identified. The ability of the fungal strain to decolorize the dye was carried out in Sabroud dextrose broth SDB amended with Malachite green dye (5 mg/L). The Erlenmeyer flasks contained 100 mL sterile media with dye and were inoculated with an immobilized fungal strain. The flasks were placed in an incubator shaker for 72 h at 30 ± 2 °C. The samples were withdrawn aseptically at 24, 30, 36, 48, and 72 h alternately and centrifuged at 4500 rpm for 10 min. Furthermore, the supernatant was scanned in a spectrophotometer at λmax (620 nm) of Malachite green dye. The control flasks underwent similar former conditions, but without fungal biomass. Among the isolated strains, *Mucor* sp. optimally decolorized Malachite green, with a removal efficiency of 92.2%. The resultant sequence was given to the National Center for Biotechnology Information (NCBI), where it was assigned an accession number (ON934589.1). Figure 2 shows that the gene sequence was examined using NCBI’s Basic Local Alignment Search Tool (BLAST) and that a phylogenetic tree was formed using Mega 7.0.

### Immobilization of *Mucor *sp. ON934589.1 in alginate

A sodium alginate stock solution prepared using 2 g of sodium alginate (R&M Chemicals) was dissolved in 50 mL of distilled water. Separately, bentonite was made by dissolving 1 g of bentonite and 1 g of active carbon in 50 mL of distilled water and stirring the mixture to create a homogeneous suspension. Afterward, the bentonite solution and alginate were combined and autoclaved for 20 min at 121 °C. A total of 10 g pellets of fungal cells were obtained through centrifugation (46,000 rpm for 21 min) after they were cultured in Sabroud dextrose broth. They were then combined with alginate (2% by weight) and bentonite (1% by weight) and dropped separately into 100 mL of CaCl_2_ solution (3% by weight) with continuous stirring. The beads formed were left for 1 h at 37 °C, washed thoroughly in distilled water, and stored for 24 h at 4 °C.

### Optimization of MG decolorization using Box–Behnken design

The Box–Behnken design was used to examine the effects of four significant variables on the decolorization of MG by immobilized *Mucor* sp. These variables included pH (5–9) (A), temperature (25–45 °C) (B), fungal concentration (1.0, 2.0, and 3.0 g), contact time (24–72 h) (C), and initial concentrations (5–200 mg/L) (D). Flasks were kept in an incubator shaker at 120 rpm, and the optical density at λ_max_ (620 nm) was recorded to determine the concentration of MG in the supernatant.

### Model verification using the experimental data

Data were analyzed using a variety of statistical techniques, including the root mean square error (RMSE), which was calculated according to Eq. (4), where n and p are the number of experimental data and parameters number of the model, respectively. Where Pdi and Obi are predicted values and experimental data, respectively. The models for describing the maximum growth rate of *Mucor* sp. were evaluated using both the bias factor (Bf) and the accuracy factor (Af), as calculated according to Eqs. (5) and (6). A model is considered fail-safe if its Bf value is more than 1.0 and fail-dangerous if its Bf value is less than 1.0. On the other hand, the value of Af is never larger than 1.0, with accurate models being characterized by values for this parameter that are close to 1.0. The Akaike information criterion (AIC) is a measure of the relative quality of mathematical analyses for a given set of data, and a criterion for error prediction was calculated according to Eq. (7). The R^2^ formula is modified for nonlinear models to incorporate the residual mean squared error and S^2^y, which is the total variance of the Y-variable^36^.

4 $$\mathrm{RMSE}=\sqrt{\sum_{i-1}^{n}\left(\frac{\mathrm{experimental}/\mathrm{predicted}}{n-p}\right)2},$$

5 $$\mathrm{Bf}= 10exp[\mathrm{ln}10 [\sum \mathrm{log}((\mathrm{experimental}/\mathrm{predicted})/n)] ],$$

6 $${\text{Af}} = { }10exp\left[ {{\text{ln}}10{ }} \left[\sum \left. {\left| {{\text{log}}\left( {\left( {\frac{{{\text{experimental}}/{\text{predicted}}}}{n}} \right)} \right.} \right|} \right)\right] { } \right],$$

7 $${\text{AICc}} = {\text{2p }} + {\text{ nLN}}\left( {{\text{RSS}}/{\text{n}}} \right) + {2}\left( {{\text{p}} + {1}} \right) + \left( {{2}\left( {{\text{p}} + {1}} \right)\left( {{\text{p}} + {2}} \right)/{\text{n}} - {\text{p}} - {2}} \right),$$

8 $$\mathrm{Adjusted }\left(\mathrm{R}2 \right)= 1\frac{RMS}{S2y},$$

9 $${\text{Adjusted }}\left( {{\text{R2}}} \right)\, = \,{1}\, - \,({1}\, - \,R{2})\left( {{\text{n}}\, - \,{1}} \right)/\left( {{\text{n}} - {\text{p}} - {1}} \right).$$

## Results and discussion

### Characterization of nano-bentonite and MgO-impregnated clay

#### XRD patterns of nano-bentonite and MgO-impregnated clay

**Figure 3:**
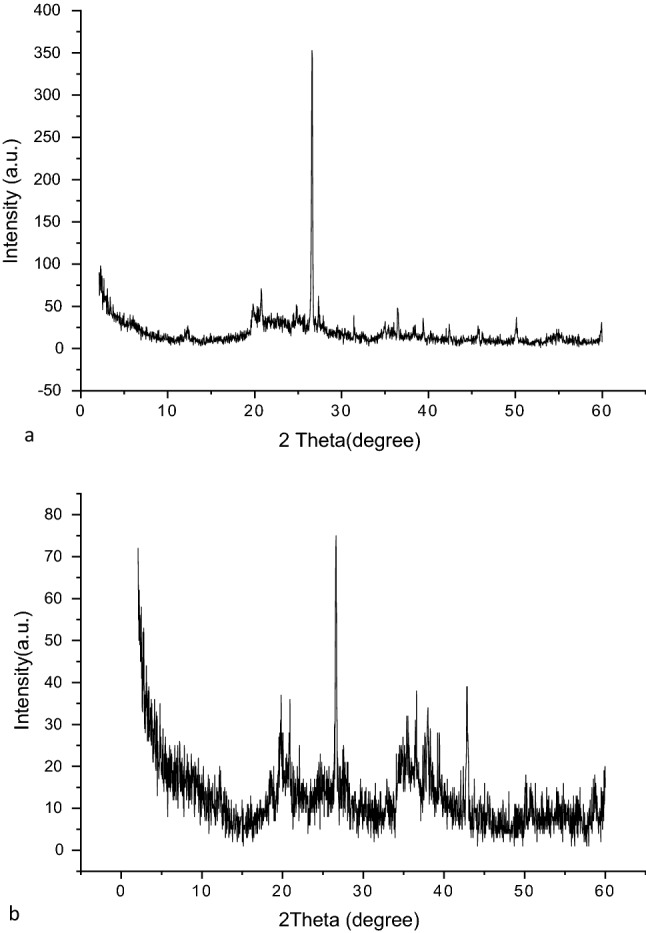
(**a**) Nano bentonite and (**b**) MgO impregnated clay XRD chromatogram after adsorption.

An XRD analysis (Fig. 3a) was conducted to determine the mineralogical constitution and crystalline nature of the nano-bentonite sample. The intensities of the XRD peaks were relatively high, which is an indication of high crystallinity. Based on the XRD pattern, we can conclude that Kaolinite-1A and quartz were the major constituents of modified bentonite, a conclusion confirmed by standard data for bentonite (ref’s: 01-075-8320 and 00-058-2028). The dominant diffraction peaks for nano-bentonite were found at values for Bragg’s angle (2θ) of ~ 12.2°,20.79°, 26.60°, ~ 27.3°, 34.88°, and. 39.43°,which are due to the presence of kaolinite, and of 19.79°, 36.47°, 42.4303°, 45.7659°, and 50.107°,which are due to the presence of quartz. The decrease of the interlayer space of nano bentonite indicates that some molecules of MG were adsorbed on top of the layers, a phenomenon that may be due to an electrostatic interaction between the positively charged groups of dye surfactant molecules with the negatively charged surface sites of nano-bentonite^37,38^. Scherrer's Eq. (10) has been used to calculate the crystallites' size (D):

10 $$D=\left(\frac{\mathrm{k\lambda }}{\upbeta }\mathrm{cos\theta }\right),$$

where D is the crystallite size, β is the full width at half maximum, λ is the X-ray wavelength, and θ is Bragg’s angle. The estimated size of the average nano-bentonite crystallite was ~ 38 nm. In Fig. 3b are reported the XRD patterns of MgO-impregnated clay. According to this figure, the said clay sample exhibited various peaks of different intensities. Indeed, peaks were observed at 2θ values of 20.91°, 26.61°, 36.57°, 37.63°, 50.14°, 56.72°, 12.27°, 18.60°, 58.76°, and 42.8392°, indicating the presence in the sample of quartzite (40%), kaolinite (10%), and MgO nanoparticles (50%), respectively. The average crystallite size was estimated to be ~ 46.6 nm. The peaks in the XRD pattern of MgO-impregnated clay generally vanished and were reduced in size, and the clay's structure changed from crystalline to slightly amorphous, demonstrating the occurrence of chemisorption processes^3^.

#### FTIR spectra of nano-bentonite and MgO-impregnated clay

**Figure 4:**
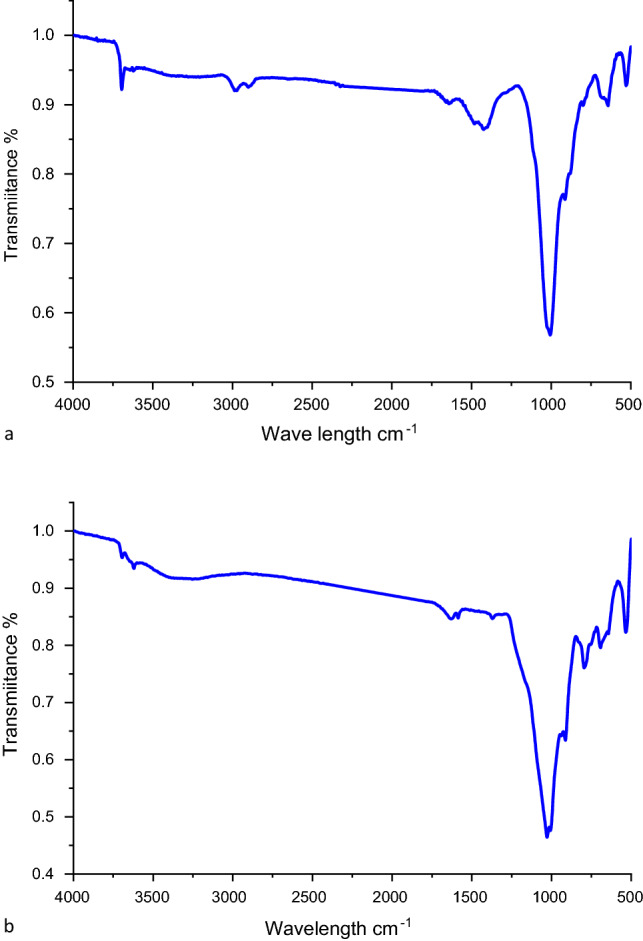
FTIR Images of nano bentonite (**a**) and (**b**) MgO impregnated into clay after MG adsorption.

The broad infrared spectroscopy bond-stretching peak between 3693.93 and 1630.21 cm^−1^ wave numbers (Fig. 4a) is indicative of the presence of OH stretching in hydration water on the bentonite surface. Notably, in Ref.^39^ detected peaks on the bentonite surface at 3450 and 1650 cm^−1^ wave numbers, which confirmed the existence of OH groups. In the FTIR spectra recorded in the present study. The stretching vibration of the Si–O bond was detected as avery strong absorption band at 1006 cm^−1^, providing strong evidence of the presence of a silicate structure. Due to the electrostatic attraction between the bentonite Si–O groups and the MG's positively charged moiety, and it indicates that the Si–O groups of bentonite may be involved in the process of dye adsorption, while the shift in the wave number values of the peaks indicates that substrate adsorption did indeed occur^40^. The peak at 920.80 cm^−1^ is attributed to the bending vibration of Al–OH–Al groups^41^. The presence of quartz in bentonite may be inferred from the peaks at 795 and 533 cm^−1^. According to^42^, the presence of quartz is confirmed by a band appearing at 796 cm^−1^. Reference^43^ attribute the bands at 500–400 cm^−1^ wave numbers to the bending vibrations of the Al–O–Si (octahedral Al) and Si–O–Si (tetrahedral Si) groups. The FTIR spectrum of the species obtained after MgO-impregnated clay underwent MG adsorption is reported in Fig. 4b. The bands at 3861 and 3622 cm^−1^ correspond to the stretching vibrations of the O–H bond of Si–OH groups coordinated to two Al atoms, whereas the band at 3207 cm^−1^ is due to MG captured by MgO. The band at 1641 cm^−1^ is due to the bending of water molecules, and the peak at 1423 cm^−1^ is attributed to the Si–O bond vibration mode. The deep band at around 1040 cm^−1^ is due to the stretching of the Si–O bond in the Si–O–Si groups of the tetrahedral sheet. The peak at 913 cm^−1^ is due to the deformation of the Al–Al–OH group; indeed, this peak is very close in position to the peaks at 913 and 914 cm^−1^ reported by Ref.^44^.The FTIR peaks appearing at 800 and 620 cm^−1^ are associated with Al–O + Si–O bending vibrations, while the peak at 537 cm^−1^ is associated with the bending vibration of the Al–O–Si group, and their observation is indicative of the presence of crystalline quartz.

#### TEM and SEM analyses

**Figure 5:**
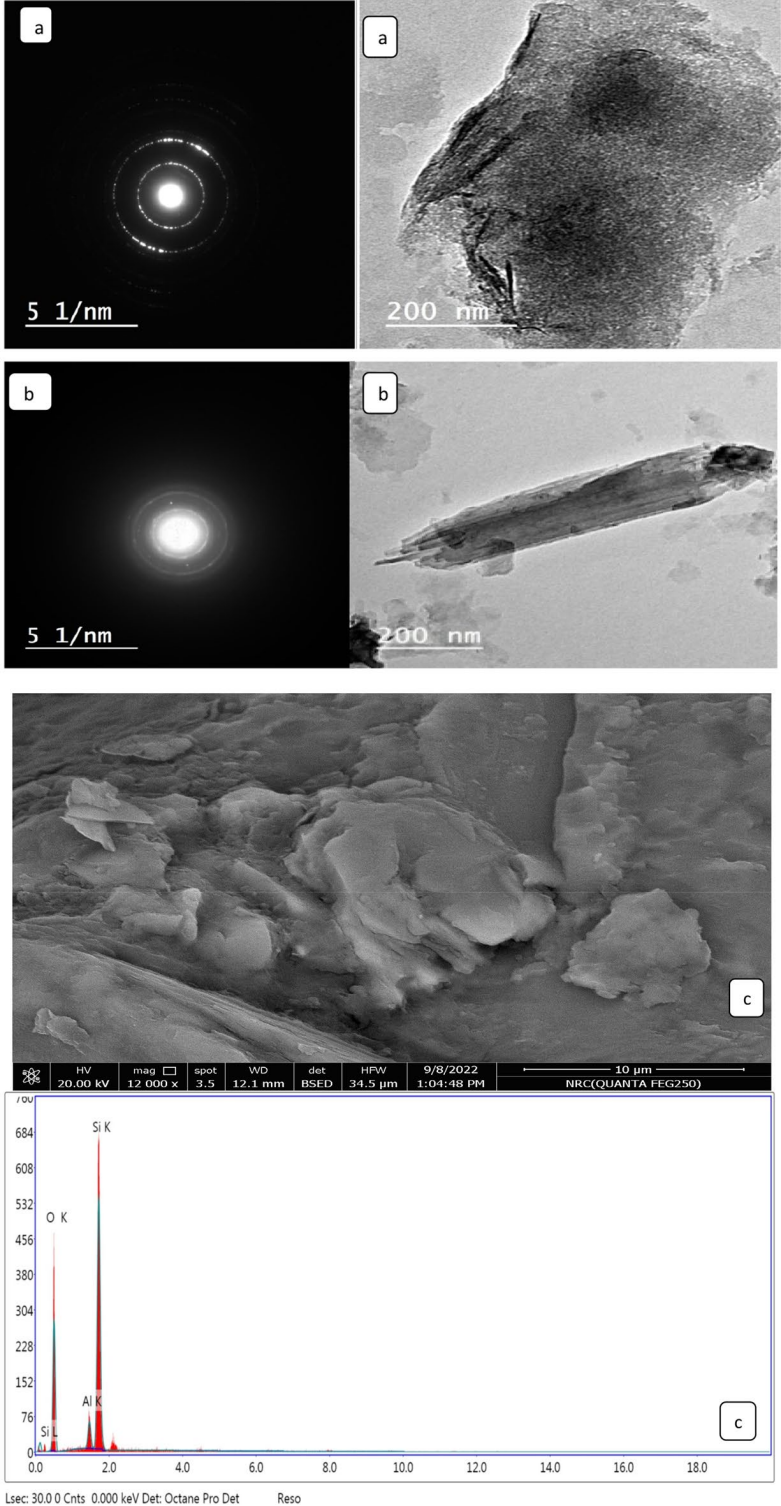

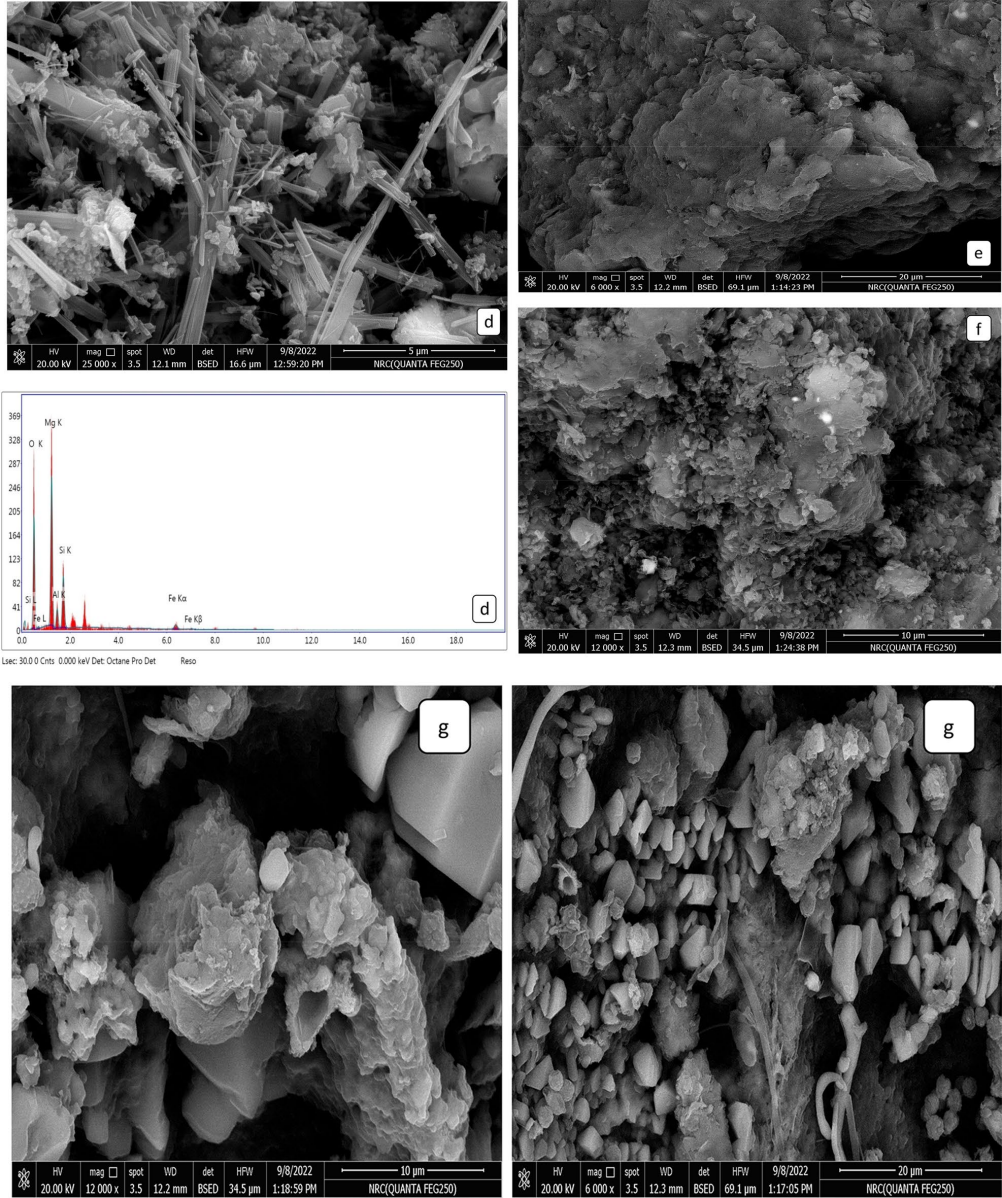
TEM (**a**) nano bentonite, (**b**) MgO impregnated clay, and SEM images and Energy dispersive X-ray analysis (**c**) nano bentonite within EDX, (**d**) MgO impregnated clay within EDX (**e**) nano bentonite after adsorption MG, (**f**) MgO impregnated clay after adsorption MG and (**g**) after adsorption MG by fungi low and high magnification respectively.

As can be evinced from Fig. 5a,b, the TEM images of nano-bentonite and MgO-impregnated clay indicated that these samples were irregularly shaped, heterogeneous, and semi-spherical. The surface morphologies of nano-bentonite and MgO-impregnated clay were investigated by SEM (see Fig. 5c,d, respectively). Nano-bentonite was observed to have a smooth surface and an irregular shape while surface morphology reveals a spongy appearance with an uneven structure. Additionally, micrographs of the MgO-impregnated clay powder indicate the presence of huge agglomerates of extremely fine MgO particles; these data also suggest that the said powder is highly porous. The generation of pores and voids may be caused by the bentonite clay swelling upon treatment with magnesium salt, which, upon desiccation and calcination, results in the formation of MgO clusters in the interlayer spaces of bentonite. At various magnifications, secondary electron images were acquired in order to study their morphologies and elemental compositions. The SEM image of the nano-bentonite and MgO-impregnated clay after adsorption of MG dye shows that the surface of the adsorbent is rough with an increased number of voids, as shown in Fig. 5e,f, respectively. The average crystallite sizes of MgO-impregnated clay and nano-bentonite, which were estimated via the Debye–Scherrer equation, were 46.6 and 38.9 nm, respectively, and were found to be close to the average particle size computed from individual particles: 43.2 and 34 nm, for MgO-impregnated clay and nano-bentonite, respectively. Figure 5g displays the surface morphologies of the fungus hyphae and active carbon after they have absorbed the MG. The outer surface of the fungal biomass and active carbon (AC) are coated with particles with diameters ranging from 0.1 to 1 mm, suggesting that the dyes were primarily adsorbable onto the fungus hyphae and AC. The presence of polysaccharides in the fungal biomass cell wall gives the hyphae ball a great capacity for biosorption^45^.

### Influence of pH on MG adsorption

**Figure 6:**
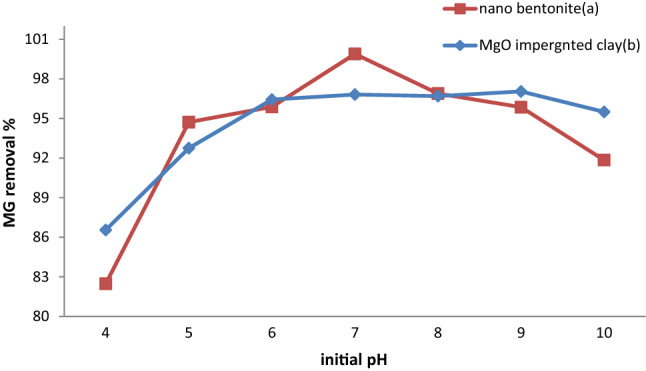
Effect of pH on MG dye removal by (**a**) nano bentonite and (**b**) MgO impregnated clay.

According to several studies, the initial pH of a solution is one of the most important environmental factors influencing the adsorption process, because it affects adsorbate solubility and surface charge, as well as adsorbate speciation and degree of ionization. The effect of the initial pH on the capacity of nano-bentonite and MgO-impregnated clay to adsorb MG was investigated over the 3.0–11.0 pH range. In Fig. 6 are reported data reflecting the influence that the initial pH had on dye removal, in conditions where by the initial concentration of the dye (50 mg/L), the contact time (60 min), the temperature (35 °C), and adsorbent dosage(1.0 g) were kept constant. As can be evinced from Fig. 6, MgO-impregnated clay exhibited good adsorption, with the maximum percent removal of MG (97.04%) observed at pH 9.0.On the other hand, the maximum uptake of MG by nano-bentonite reached a value of 99.8% at pH7.0. The values for the zero point charge (pHzpc) of nano-bentonite and MgO-impregnated clay were found to be 5.5 and 7.1, respectively. Thus, at pHzpc (5.5 and 7.1, respectively), the nano-bentonite and MgO-impregnated clay had net positive surface charge and negative surface charge at pH > pHpzc. The low adsorption capacity exhibited by the two species under acidic conditions could be mainly attributed to the decrease in the number of negative charges on the adsorbents’ surfaces and the increase in the number of positively charged sites in the adsorbents, which can cause electrostatic repulsion between the adsorbent and the dye molecules; moreover, the presence of excess amounts of H^+^ ions may result in the said ions competing with the cationic MG species for adsorption onto nano-bentonite and MgO-impregnated clay. As a consequence, the probability of MG molecules being adsorbed on the two adsorbents may decrease. By contrast, as the pH increased, the deprotonation of the acid sites on the surface of nano-bentonite and MgO-impregnated clay composites resulted in the number of negatively charged adsorbent sites to increase^46^. According to Ref.^47^, who examined the relationship between pH and the adsorption of MG onto bentonite, the interactions between the cationic amine moiety of MG and the negatively charged SiO_2_ in the bentonite. The cationic active sites are present and exhibit an increased likelihood of binding MG when the pH of the solution is between 5 and 6. As a result of the strong electrostatic interactions between MG and the adsorbents, the surface diffusion of the dye molecules increases. Similar conclusions were reached by Ref.^30^,who attributed the increase in adsorption observed as the pH increased to a reduction in the competition for functional groups between the target cations and the protons present in solution. Our findings paralleled those of Ref.^48^, who discovered that the removal of MG dye by titanium coated graphite was lowest at pH 3.0 (56.2%) and highest at pH 7 (95%). Our results are consistent with those reported in Ref.^17^ at pH 7, the Shell's seeds of *Ziziphus spina christi* adsorbed 91.1% of Malachite green dye.

### Influence of the temperature on MG adsorption

**Figure 7:**
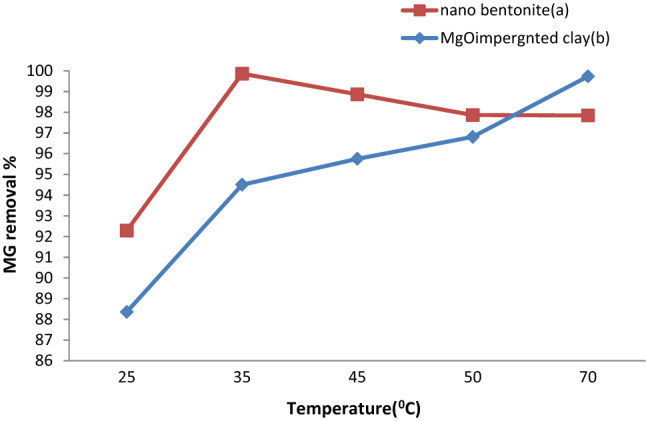
Effect of temperature on MG dye removal by (**a**) nano bentonite and (**b**) MgO impregnated clay.

One of the factors that was observed to affect MG adsorption is the temperature. The effect of temperature on dye discoloration was evaluated preparing a mixture of MG with nano-bentonite or MgO-impregnated clay at different temperatures in the 25 °C–70 °C range, while keeping the adsorbent dosage (0.7 g), the pH (7), the contact time (60 min), and agitation speed (200 rpm) constant. As can be evinced from Fig. 7, evidence indicates that the rate of adsorption of MG onto nano-bentonite increased as the temperature was raised from 25 to 35 °C; the said rate of MG adsorption then gradually decreased as the temperature rose above 40 °C, an observation that may be attributed to the bonds between the dye molecules and the active sites of the adsorbents getting weakened. At 25 °C, the percentage removal of MG was 92.2%; at 35 °C, this parameter increased to 99.8%; past the said temperature mark, the percent MG removal did not display any significant change until the temperature reached 70 °C.This evidence indicates that the adsorption process was slightly endothermic. A similar trend was reported by Ref.^28^ for the removal methylene blue by activated bentonite. On the other hand, the percent removal of MG by MgO-impregnated clay increased when the temperature was raised from 25 to 70 °C. At 25 °C, the percentage removal of the dye was 88.3%; at 70 °C, this value increased to 99.7%. The adsorption process was exothermic, as shown in Fig. 7. Indeed, the increase in percentage removal is observed because the kinetic energy of the molecules increases as the temperature increases, and the accelerated molecules disperse faster in the adsorbent^49^. In addition, the increased temperature will cause the internal structure of the adsorbent to swell, allowing large dyes to penetrate the adsorbent^50^. This finding was consistent with the data reported by Saleh Bashanaini^17^, who found that the value for MG removal with activated carbon prepared from shells seeds increased up to 95% as a result of the temperature being raised to 50 °C.

### Influence of contact time on MG adsorption

**Figure 8:**
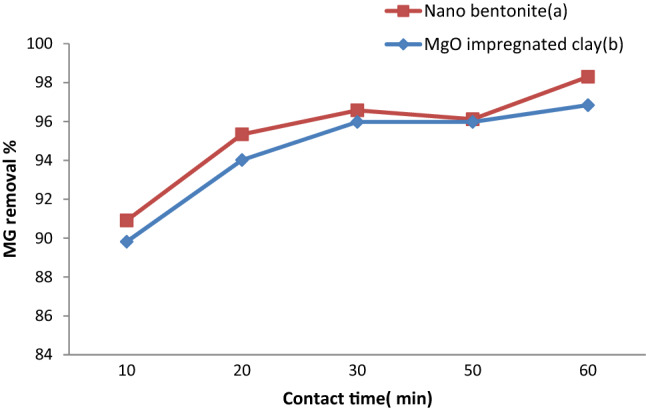
Effect of contact time on MG dye removal by (**a**) nano bentonite and (**b**) MgO impregnated clay.

Assessing the effect of the contact time is important because the results of such an investigation provide basic information on how quickly the adsorption process reaches equilibrium. The effect that changing the contact time in the 10–60 min range had on the adsorption capacity was studied while other parameters were kept constant (adsorbent dosage, 0.7 g; pH 7; initial MG concentration, 50 mg/L; agitation speed, 200 rpm; temperature, 35 °C). Based on the results reported in Fig. 8, rapid dye adsorption was observed in the initial phase of the experiment; subsequently, dye adsorption gradually slowed down, as the equilibrium condition was approached after about 60 min. In the case of nano-bentonite, at the 10 min mark, the percentage removal of MG was 90.9%, and the value for this parameter gradually increased to 95.3% at the 20 min mark and to 98.2% at the 60 min mark. In the case of the MgO-impregnated clay, the MG percentage removal was89.8% at the 10 min mark; it increased sharply to 95.9% at the 30 min mark, and to 96.8% at the 60 min mark. In fact, the maximum removal efficiency achieved by nano-bentonite was 98.2%, where as that achieved by MgO-impregnated clay was 96.8%.The time required to reach equilibrium in the adsorption of MG on nano-bentonite and on MgO-impregnated clay was found to be 30 min. The described trend could be rationalized by envisioning a situation where by MG molecules proceeded to occupy a large number of initially vacant active sites onthe surfaces of the adsorbents, resulting in a high initial adsorption rate; as the contact time increased, however, the MG adsorption rate decreased as the number of vacant sites decreased and the repulsive forces between the dye molecules adsorbed on the biomass increased, and the large phase led to a significant decrease in absorption capacity, so that the dye molecules slowly diffused into the interior of the adsorbents^51^. The results of the present study are consistent with those reported by Tarekegn and Balakrishnan^3^ on the effects of contact time on the adsorption of methylene blue dyes on nano-zero-valent iron, nano-clay, and iron-impregnated nano-clay^52^. Present study is consistent with previous literature^3^ who, reported that the effects of contact time on the adsorption of Malachite green dye on the titanium coated graphite with CNT-ABS adsorbents achieved removal efficiency at 35%, (20 min) and it increased to 97.3% at 60 min.

### Influence of the initial MG concentration on dye adsorption

**Figure 9:**
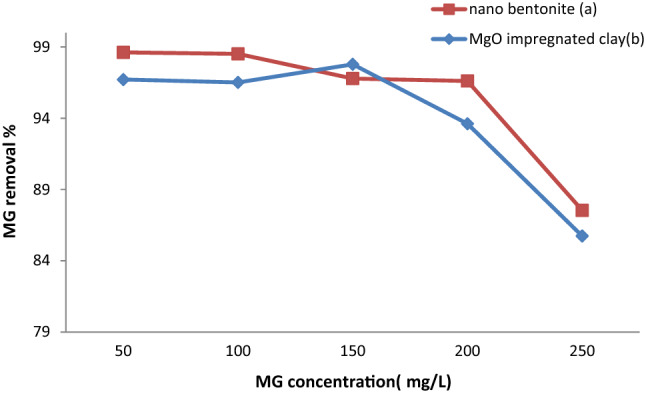
Effect of initial MG dye concentration on MG dye removal by (**a**) nano bentonite and (**b**) MgO impregnated clay.

The effect of the initial concentration of MG on nano-bentonite and MgO-impregnated clay was investigated by making the said concentration vary in the 50–250 mg/L, range, while the other parameters were kept constant (contact time, 60 min; pH 7; initial concentration, 50 mg/L; agitation speed, 200 rpm; temperature, 35 °C). The dye removal efficiency of the adsorbents declined as the initial concentration of MG increased. Notably, the dye adsorption activity of MgO-impregnated clay was less influenced by changes in the initial concentration of the adsorbate than nano-bentonite. The MG removal efficiency of MgO-impregnated clay declined from 96.7 to 89.7% as the initial MG concentration increased from50 to 250 mg/L (see Fig. 9). While nano-bentonite achieved a maximum MG removal efficiency of 98.6% at an initial concentration of MG of 50 mg/L, this parameter’s value was reduced to 91.5% when the initial concentration of MG increased to 250 mg/L. This trend can probably be explained considering that the lower the initial concentration of MG, the larger the proportion of initially vacant (available) active sites on the surface of the adsorbent. Fairly similar observations were reported by Ref.^2,48^. Our results were in agreement with the previous study by Ref.^3^, which found that the iron impregnated clay's ability to remove MB dye from 98.86 to 76.80% at doses of 20–80 mg/L, respectively.

### Influence of adsorbent dosage on MG adsorption

**Figure 10:**
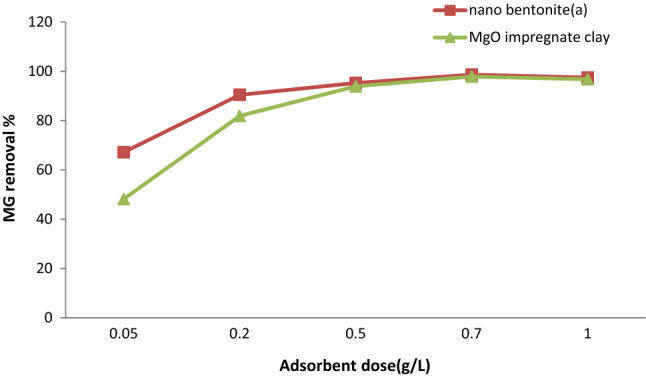
Effect of adsorbent dose on MG dye removal by (**a**) nano bentonite and (**b**) MgO impregnated clay.

The dosage of nano-bentonite and MgO-impregnated clay was another crucial factor influencing dye adsorption activity. The dosages of nano-bentonite and MgO-impregnated clay were made to have the following values: 0.1, 0.2, 0.5, 0.7, and 1.0 g; in the experiments conducted, the initial MG concentration (50 mg/L), temperature (35 °C), agitation speed (200 r/min), and pH (7) were kept constant. As can be evinced from in Fig. 10, the dosages of nano-bentonite and MgO-impregnated clay that afforded the maximum rate of MG removal were 1.0 and 0.7 g, respectively. The maximum MG removal efficiency achieved by nano-bentonite was 98.6% and that achieved by MgO-impregnated clay was 97.4%. The MG adsorption rate of MgO-impregnated clay increased sharply from 48.1% measured at a 0.05 g dosage of MgO-impregnated clay to 97.8% at a 0.7 g dosage of the said adsorbent. The adsorption efficiency of MgO-impregnated clay then increased gradually to 98.1% at a 1.0 g dosage. By contrast, nano-bentonite exhibited a higher adsorption rate than MgO-impregnated clay at a 0.05 g dosage (67.1%) and a more gradual increase in adsorption efficiency as its dosage increased to 0.7 g (adsorption rate: 99.8%). The MG removal efficiency remained constant as the adsorbent dosage was made to increase further to 1.0 g (Fig. 10). The results of these experiments were consistent with those reported by Ref.^53^, which indicated that the initial concentration of MG was inversely proportional to the efficiency of MG adsorption on silver nanoparticles coated on activated carbon.

### Description and analysis of the quadratic model

In order to optimize the adsorption process, a Box–Behnken design with four factors (initial concentration of dye, temperature, adsorbent dose, and pH) was chosen. The higher and lower levels of the variables are listed in Table 1, while the experimental and predicted values of the percentage decolorization of MG in the presence of nano-bentonite and MgO-impregnated clay are listed in Table 2. The second-order response surface polynomial function (Eqs. 11, 12) can be used to predict the dye's optimum operating circumstances:

11 $$\begin{aligned} {\text{Y}}({\text{nano - bentonite}}) & = 97.3092 + 2.839 \times {\text{A}} + - 0.72275 \times {\text{B}} + 0.0690833 \times {\text{C}} + - 0.0521667 \\ & \quad \times {\text{D}} + 0.69575 \times {\text{AB}} + - 0.67425 \times {\text{AC}} + 0.321 \times {\text{AD}} + - 1.136 \times {\text{BC}} + 0.8515 \times {\text{BD}} + \\ & \quad - 0.4435 \times {\text{CD}} + - 2.37618 \times {\text{A}}^{2} + - 0.276058 \times {\text{B}}^{2} + - 0.215808 \times {\text{C}}^{2} + 0.0813167 \times {\text{D}}^{2} , \\ \end{aligned}$$

12 $$\begin{aligned} {\text{Y}}({\text{MgO - impregnated}}\,{\text{clay}}) & = 97.4599 + 1.08619 \times {\text{A}} + - 0.952475 \times {\text{B}} + - 0.0289832 \times {\text{C}} \\ & \quad + - 0.830585 \times {\text{D}} + 0.964158 \times {\text{AB}} + - 1.15204 \times {\text{AC}} + - 1.9757 \times {\text{AD}} + 1.0302 \times {\text{BC}} \\ & \quad + 0.528359 \times {\text{BD}} + - 0.482124 \times {\text{CD}} + - 3.78607 \times {\text{A}}^{2} + - 0.135132 \times {\text{B}}^{2} + - 0.163015 \\ & \quad \times {\text{C}}^{2} + 0.188028 \times {\text{D}}^{2} . \\ \end{aligned}$$

**Table 2:** Experimental design and results of the Box Ben design for the decolourization of MG by nano bentonite and MgO impregnated clay.

Run	pH	Concentration	Temperature	Dosage	Nano bentonite	MgO impregnated clay
1	0	0	0	0	97.13	90.0855
2	− 1	0	0	− 1	92.429	91.4188
3	0	0	0	0	97.212	93.7188
4	0	0	0	0	97.114	96.6815
5	0	1	0	1	97.212	97.329
6	1	0	0	1	98.409	97
7	0	− 1	0	1	96.51	98.9544
8	0	1	1	0	96.118	97.1882
9	− 1	0	1	0	92.401	93.0563
10	0	1	0	− 1	96.016	96.412
11	1	0	0	− 1	97.392	93.8901
12	0	0	1	− 1	98.05	97.9188
13	0	− 1	0	− 1	98.72	97.0188
14	0	0	− 1	− 1	96.413	97.8514
15	0	0	− 1	1	97.119	98.8514
16	− 1	1	0	0	90.015	97.012
17	0	− 1	1	0	98.812	94.9188
18	1	0	1	0	96.47	95.8146
19	0	− 1	− 1	0	96.312	94.8188
20	− 1	0	0	1	90.062	97.512
21	− 1	− 1	0	0	92.32	94.1523
22	1	− 1	0	0	97.84	96.0521
23	1	0	− 1	0	98.382	95.029
24	0	0	0	0	97.3	92.0092
25	− 1	0	− 1	0	89.616	96.945
26	0	1	− 1	0	97.162	92.0402
27	0	0	0	0	97.8	98.512
28	0	0	1	1	96.982	99.7798
29	1	1	0	0	97.318	90.0855

**Figure 11:**
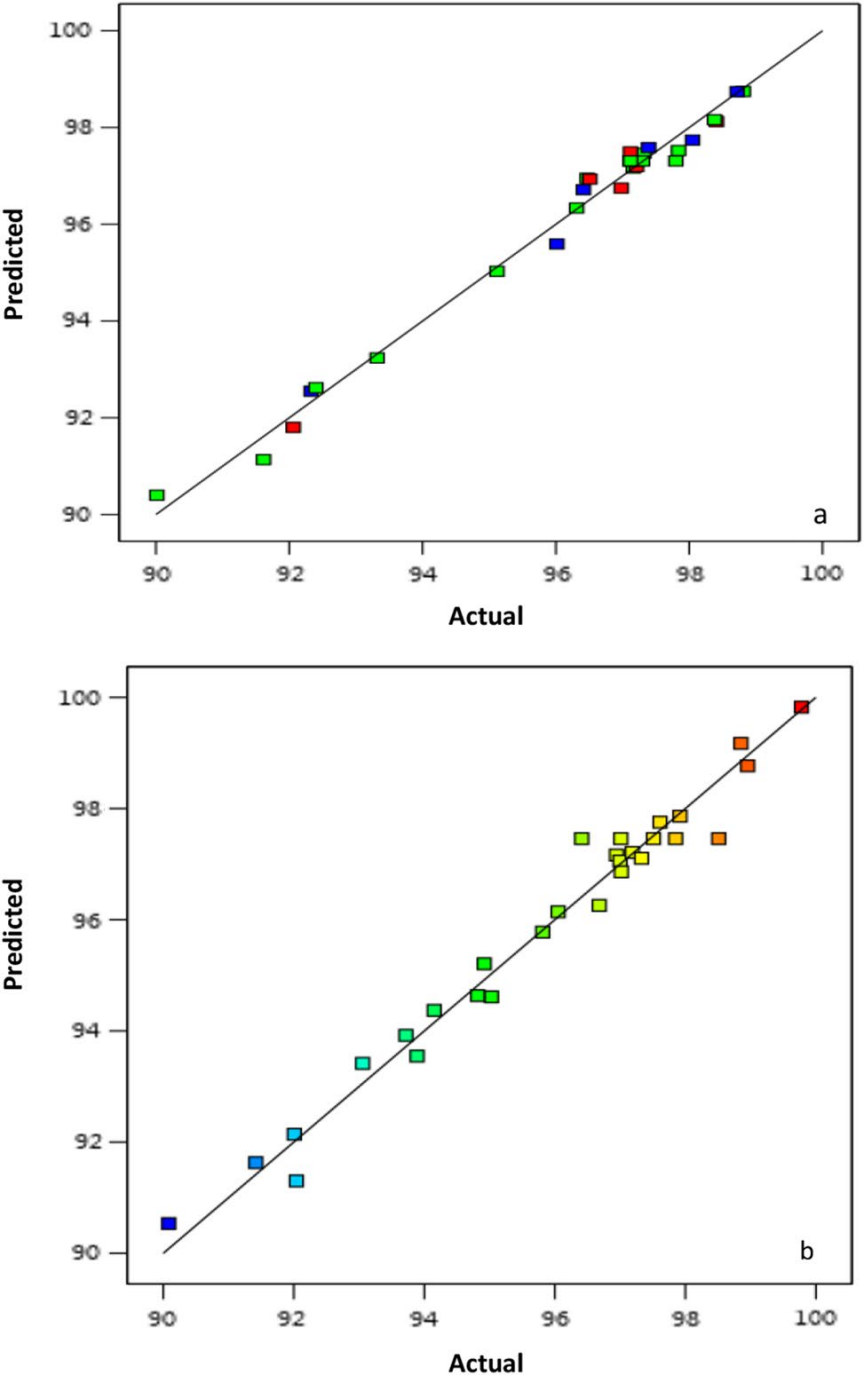
Linear correlation between experimental and predicted removal efficiency % MG by (**a**) nano bentonite and (**b**) MgO impregnated clay.

The assessment of variance (ANOVA) for MG elimination efficiency in the cases of nano-bentonite and MgO-impregnated clay was applied in order to validate the model, as given in Tables 3, 4. The correlation between the variables and the responses was determined using the quadratic model and second-order polynomial analysis. The Model F-values of MG removal percentage achieved by nano-bentonite and MgO-impregnated clay were recorded as 71.81and 36.85, respectively, which were favorable. The model P-values of both models for MG removal were acceptable. Model terms are considered significant when the P-value is less than 0.0500. In this case, A, B, D, AB, AC, AD, BC, and A^2^ are significant model terms for MgO-impregnated clay. When the value was higher than 0.1, model terms were not considered significant. On the other hand, the model F-value of nano-bentonite was 71.81, indicating that the model was favorable. In this case, A, B, AB, AC, AD, BC, BD, CD, and A^2^ were satisfied model terms. The lack of fit F-value of nano-bentonite and MgO-impregnated clay were 2.62 and 0.29, respectively, implies the lack of fit is not significant relative to the pure error. There was a 22.64% and 94.48% chance for nano-bentonite and MgO-impregnated clay, respectively that a lack of fit F-value this large could be due to noise. A non-significant lack of fit indicated that the quadratic model was fit for the present study. The second-order polynomial equation was developed based on these findings to indicate a relationship between MG elimination percentage and a number of different variables. Only 0.2% and 0.9% of the total variation could not be explained by the model, according to the regression equation derived after the ANOVA, which indicated that the correlation coefficient (R^2^) values for the MG dye removal by nano-bentonite and MgO-impregnated clay were 0.986 and 0.973, respectively. A high R^2^ value (close to1) indicates that the calculated and observed findings within the experimental range are in good agreement with each other, and it also demonstrates that an acceptable and reasonable agreement with adjusted R^2^. The predicted R^2^ values for nano-bentonite and MgO-impregnated clay were 0.929 and 0.91, respectively, which are reasonably consistent with the adjusted R^2^ values: 0.952 and 0.947, respectively. These results demonstrated the effectiveness of the established model and the accuracy and minimal inaccuracy of the independent variable values. Adequate precision is used to determine of the signal to the noise ratio. A ratio larger than 4 is desirable. The values for this ratio were 29.5 and 22.842 for nano-bentonite and MgO-impregnated clay, respectively, indicating the reliability of the experimental data. The repeatability of the model is measured using a parameter called coefficient of variation (CV%), which is the ratio of the standard error of the estimate and the mean value of the observed response (expressed as a percentage).Typically, a model is regarded as replicable if its CV% value is less than 10%^54^. According to the data listed in Tables 3 and 4, the CV% values of nano-bentonite and MgO-impregnated clay are relatively small, 0.4 and 0.5%, respectively, which indicated that the deviations between experimental and predicted values were low. The plots between experimental (actual) and predicted values of MG removal by RSM model are reported in Fig. 11a,b. Based on this figure, the average differences between the predicted and experimental values can be evinced to be less than 0.1, which indicates that most of the regression model provided an explanation for the data variation.

**Table 3:** Analysis of variance (ANOVA), results for decolourization of MG by nano bentonite.

Source	Sum of squares	df	Mean square	F-value	p-value	
Model	154.80	14	11.06	71.82	< 0.0001	Significant
A-pH	96.72	1	96.72	628.26	< 0.0001	
B-Concentration	6.27	1	6.27	40.72	< 0.0001	
C-Temperature	0.0573	1	0.0573	0.3720	0.5517	
D-Dose	0.0327	1	0.0327	0.2121	0.6522	
AB	1.94	1	1.94	12.58	0.0032	
AC	1.82	1	1.82	11.81	0.0040	
AD	0.4122	1	0.4122	2.68	0.1241	
BC	5.16	1	5.16	33.53	< 0.0001	
BD	2.90	1	2.90	18.84	0.0007	
CD	0.7868	1	0.7868	5.11	0.0402	
A^2^	36.62	1	36.62	237.90	< 0.0001	
B^2^	0.4943	1	0.4943	3.21	0.0948	
C^2^	0.3021	1	0.3021	1.96	0.1830	
D^2^	0.0429	1	0.0429	0.2786	0.6059	
Residual	2.16	14	0.1539			
Lack of fit	1.83	10	0.1831	2.26	0.2246	Not significant
Pure error	0.3243	4	0.0811			
Cor total	156.96	28				
Std. Dev	0.3924		R^2^	0.9863		
Mean	96.16		Adjusted R^2^	0.9725		
C.V. %	0.4080		Predicted R^2^	0.9296		
			Adeq Precision	29.5754

**Table 4:** Analysis of variance (ANOVA), results for decolourization of MgG by MgO impregnated clay.

Source	Sum of squares	df	Mean square	F-value	p-value	
Model	164.92	14	11.78	36.85	< 0.0001	Significant
A–A	14.16	1	14.16	44.29	< 0.0001	
B–B	10.89	1	10.89	34.06	< 0.0001	
C–C	0.0101	1	0.0101	0.0315	0.8616	
D–D	8.28	1	8.28	25.90	0.0002	
AB	3.72	1	3.72	11.63	0.0042	
AC	5.31	1	5.31	16.61	0.0011	
AD	15.61	1	15.61	48.84	< 0.0001	
BC	4.25	1	4.25	13.28	0.0027	
BD	1.12	1	1.12	3.49	0.0827	
CD	0.9298	1	0.9298	2.91	0.1102	
A^2^	92.98	1	92.98	290.86	< 0.0001	
B^2^	0.1184	1	0.1184	0.3705	0.5525	
C^2^	0.1724	1	0.1724	0.5392	0.4749	
D^2^	0.2293	1	0.2293	0.7174	0.4112	
Residual	4.48	14	0.3197			
Lack of fit	1.91	10	0.1914	0.2988	0.9448	Not significant
Pure error	2.56	4	0.6404			
Cor total	169.40	28				
Std. Dev	0.5654					
Mean	95.85					
C.V. %	0.5899					
R^2^	0.9736					
Adjusted R^2^	0.9472					
Predicted R^2^	0.9113					
Adeq Precision	22.8424

### Interpretation of variable interaction on MG removal

**Figure 12:**
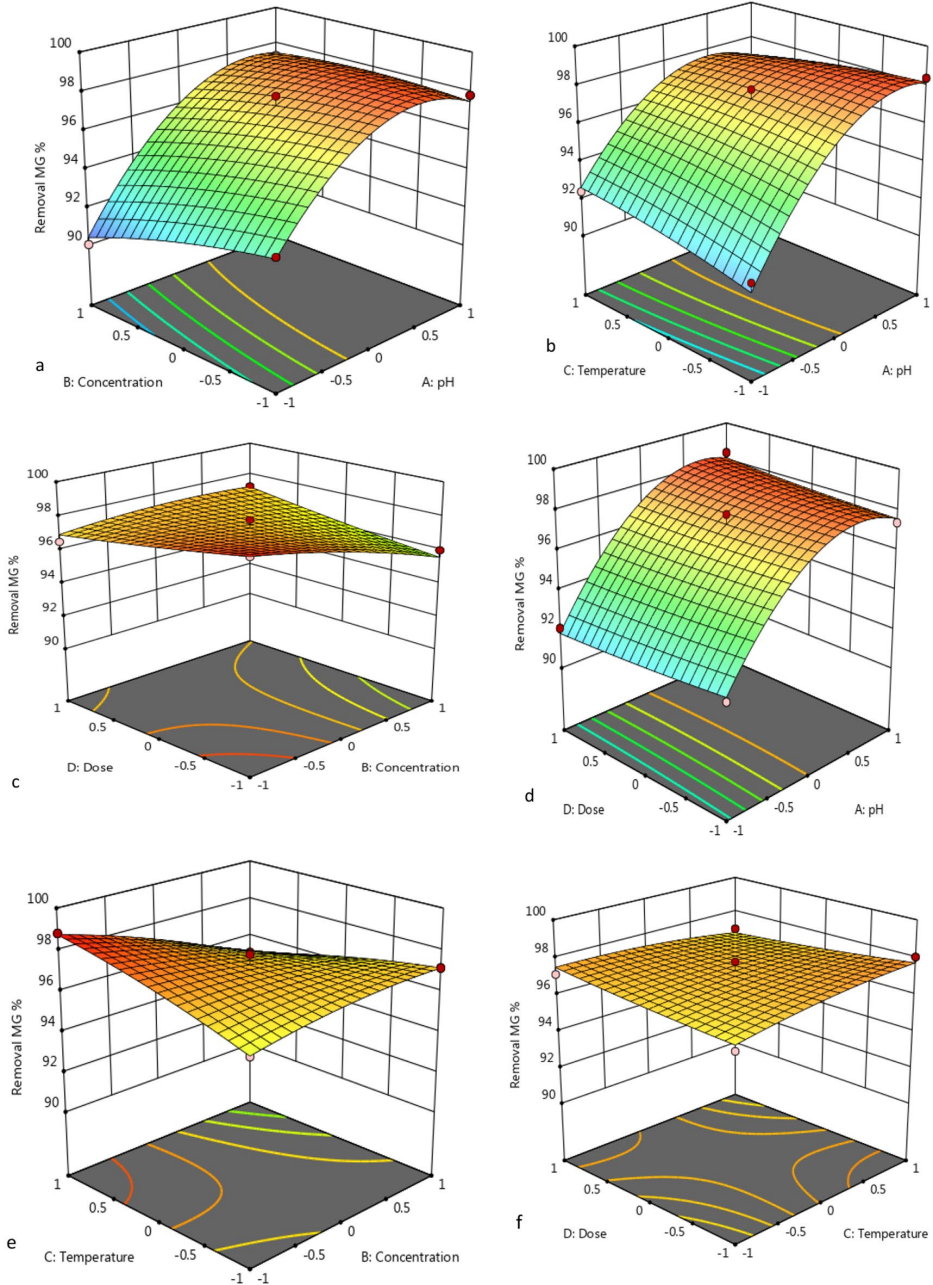
3D response surface plot of MG removal % through nano bentonite as a function of pH, temperature and adsorbent dosage.

**Figure 13:**
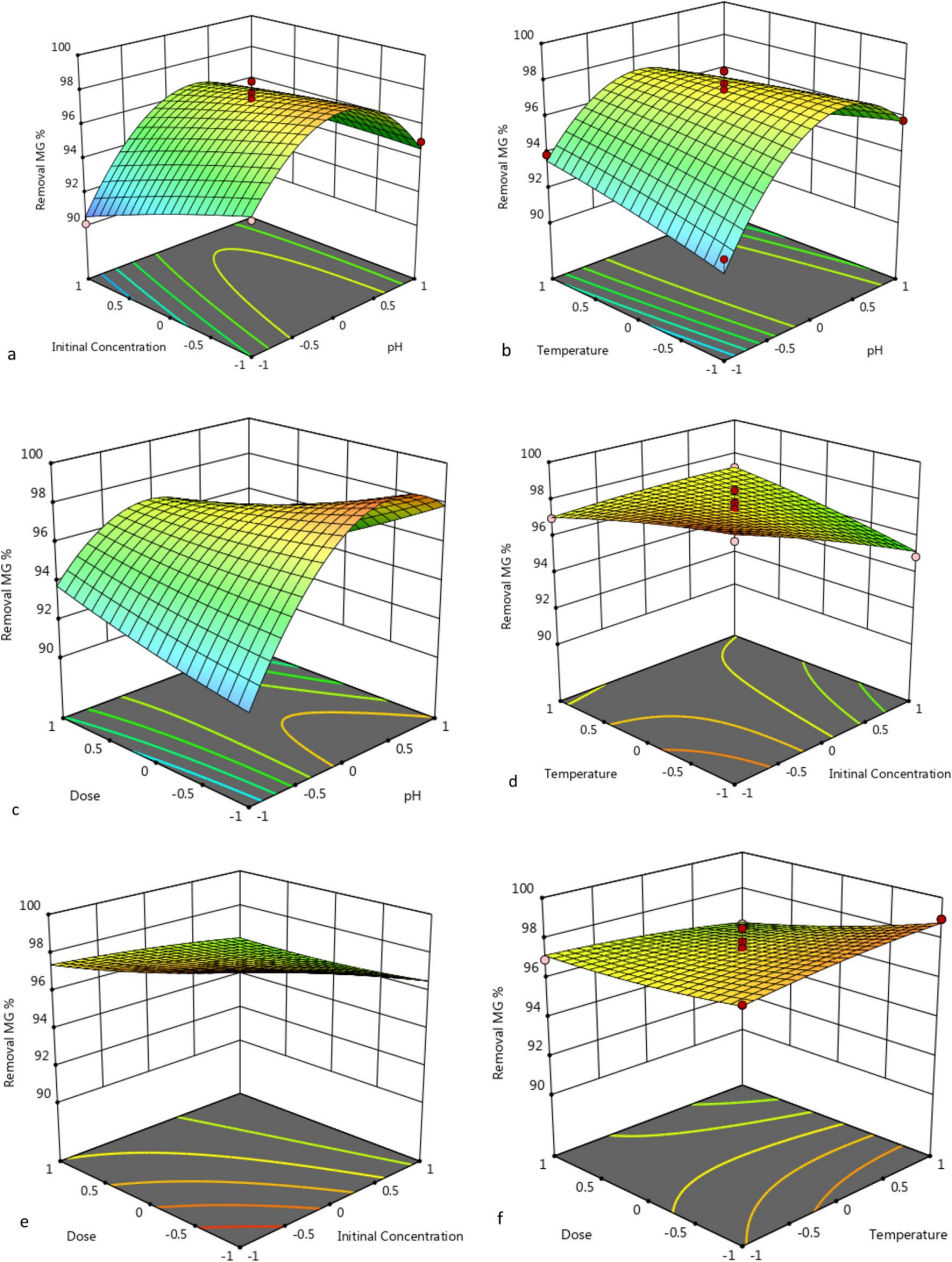
3D response surface plot of MG removal % through MgO impregnated clay as a function of pH, temperature and adsorbent dosage.

Three-dimensional surface plots and contour plots were generated to investigate the interaction between MG removal efficiency and two parameters at a time, while the other variables were held at constant values. The data reported in Figs. 12a,b and 13a,b demonstrate unequivocally that, as the temperature increased, so did decolorization percentage along with increasing pH. The maximum removal of MG dye decolorization from 25 to 35 °C, for nano-bentonite and 25–50 °C for MgO-impregnated clay, with increasing pH 7.0 and pH 9.0, there was a rise in the percentage of decolorization, respectively.

#### Effect of dose and temperature on removal of MG by nano-bentonite and MgO-impregnated clay

The elliptical shape of the curve is indicative of a high degree of interaction between the three variables. When the interaction of the MG removal efficiency with the adsorbent dosage and the adsorption temperature was examined, it was discovered that this analysis's affecting factor was temperature, as can be evinced from Figs. 12c,d and 13c,d. As the adsorbents’ dosage increased, so did the rate of MG decolorization. The temperature was found to be most influential at an nano-bentonite dosage of 0.2 g/L, in which casea79% decolorization could be observed at 25 °C and 98% decolorization could be observed at 35 °C. Maximum decolorization could be observed at a temperature of 35 °C and adsorbent dosage of 1.0 g/L. On the other hand, maximum decolorization of MG afforded by MgO-impregnated clay could be observed (97%) at a temperature of 50 °C.

#### Effect of pH and initial concentration on removal of MG by nano-bentonite and MgO-impregnated clay

The data reported in Figs. 12e,f and 13e,f reflect the effect that the pH and the initial MG concentration had on the percentage of MG removed, in conditions where by the temperature was kept constant. Above a specific initial MG concentration (above 300 mg/L), the adsorption capacity declines as the initial MG concentration increases, but there was a net positive interaction effect, suggesting that the adsorption capacity increases as the initial MG concentration and initial pH increase. The maximum capacities for MG adsorption by nano-bentonite and MgO-impregnated clay were observed at pH values in the 7.0–9.0 range. Thus, evidence indicates that the percentage of noxious dye elimination afforded by nano-bentonite and MgO-impregnated clay was very low at acidic pH 5.0.

#### Effect of dosage and initial concentration on removal of MG by nano-bentonite and MgO-impregnated clay

Referring back to Figs. 12 and 13, the combined effect on MG removal efficiency of changing the adsorbent dosage and the initial MG concentration were investigated, in conditions where by the temperature and pH were fixed at zero level. As can be evinced from Fig. 12, more than 98% and 90% of the MG dye was removed in the presence of nano-bentonite and MgO-impregnated clay under the mentioned conditions, respectively. Notably, the maximum MG removal percentage was obtained at high adsorbent dosage (0.7 g/L for nano-bentonite) and (1.0 g/L for MgO-impregnated clay), and minimum dye concentration (100 mg/L). As can be evinced from Figs. 12 and 13, dye adsorption decreased as the initial MG concentration increased. This trend may be due to the fixed number of active sites on the adsorbent vis-à-vis an increasing number of dye molecules. Banerjee and Sharma^55^ reported that the efficiency of dye adsorption on the adsorbents dropped significantly as the initial adsorbate concentration increased.

### Kinetic adsorption for MG removal

Kinetic studies on the adsorption of MG onto nano-bentonite and MgO-impregnated clay were conducted by fitting the experimental data with pseudo-1st-order and pseudo-2nd-order reaction rate equations.

#### Pseudo-first-order kinetics fitting of MG adsorption data

**Figure 14:**
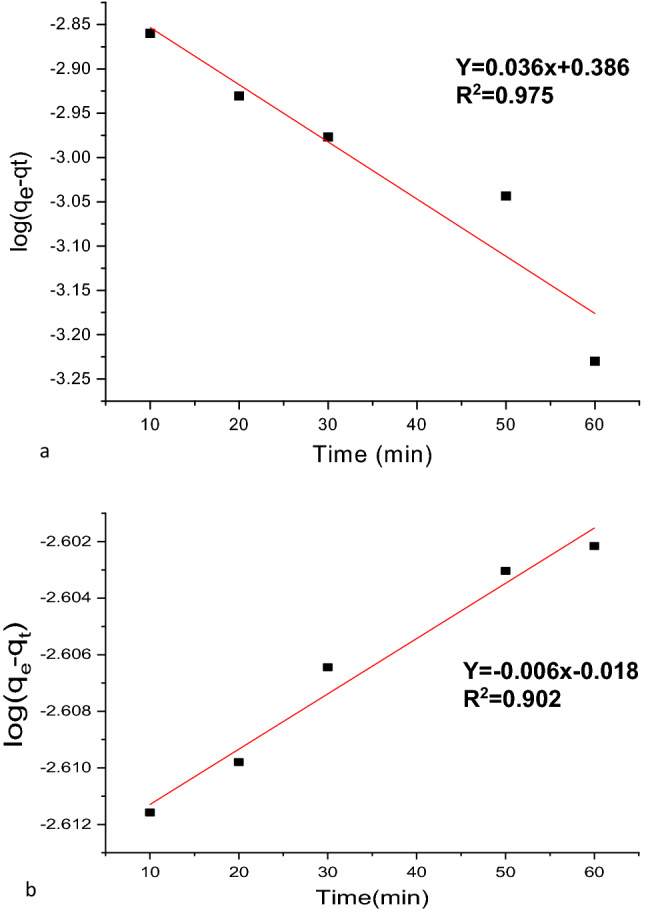
Pseudo-first order for the adsorption of MG onto (**a**) nano bentonite and (**b**) MgO impregnated clay.

The experimental kinetic data were fitted with the Lagergren pseudo-first-order rate equation (Eq. 13)^56,57^:

13 $$\mathit{log}\left({q}_{e}-{q}_{t}\right)=\mathit{log}{q}_{e}-\left(\frac{{k}_{1}}{2.303}\right)t,$$

where k_1_ is the pseudo-primary order rate constant (min^−1^), q_e_ represents the amount of MG removed at time-point t (min) of adsorbent (mg/g), and q_t_ represents the MG adsorption capacity at equilibrium (mg/g). In Fig. 14a,b is reported the plot of log (q_e_ − qt) versus time, whereas the relevant R^2^ values and constant quantity for such various adsorption kinetic designs are listed in Table 5. Given the discrepancy between the calculated (q_e_, cal) and experimentally determined (Expq_e_) adsorption capacities, which can be evinced from Table 5, a pseudo-first-order kinetics model was unable to explain the adsorption of MG onto nano-bentonite and MgO-impregnated clay. Additionally, when compared to the pseudo-second-order value, the values of the coefficient of determination (R^2^) were relatively small at 0.975 and 0.916, for the nano-bentonite and MgO-impregnated clay cases, respectively.

**Table 5:** Kinetic parameters for the adsorption MG onto nano bentonite and MgO impregnated clay.

Model	Kinetic parameter	Nano benonite	MgO-clay
Pseudo first-order	qe1 cal (mg/g)	2.4	1.01
	K1 (min^−1^)	0.08	6.5
	R^2^	0.975	0.91
Pseudo second-order	qe2. (mg/g)	0.398	0.00001
	K2 (min^−1^)	0.016	0.003
	R^2^	0.996	0.999

#### Pseudo-second-order kinetics fitting of MG adsorption data

The equation for the Lagergren pseudo-second-order kinetics (Eq. 14) is stated linearly as shown below^2,57^:

14 $$\frac{t}{{q}_{t}}= \frac{1}{{k}_{2}{q}_{e}^{2}}+ \frac{1}{{q}_{e}}\mathrm{t},$$

where k_2_ is the pseudo-second-order rate constant of MG adsorption (g/mg^/^min), and t is the contact time (min). The fitness of the straight line (R^2^) and the consistency between the experimental and calculated values of q_e_ serve as indicators of each model's validity.

**Figure 15:**
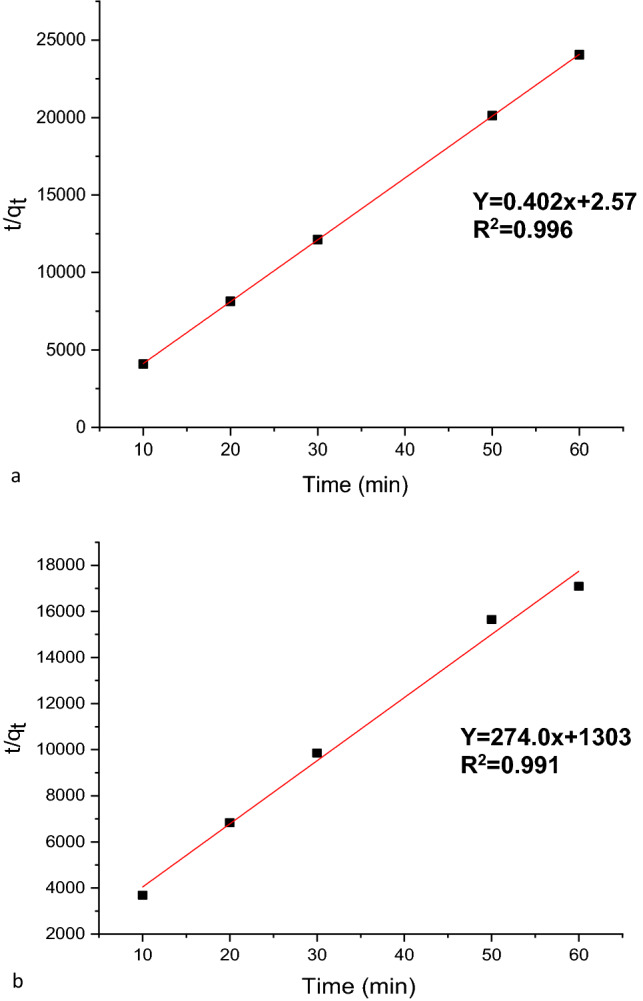
Pseudo-second order for the adsorption of MG onto (**a**) nano bentonite and (**b**) MgO impregnated clay.

The plot of t/q_t_versus the contact time is reported in Fig. 15a,b, and the values for the relevant parameters (R^2^, slope, intercept, pseudo-first order rate constant) "K1", and the experimental and calculated dye uptake levels) are listed in Table 5. As can be evinced from this table, the R^2^ values for nano-bentonite (0.996) and MgO-impregnated clay (0.999) were quite close to 1. The computed q_e_ values for both nano materials were in excellent agreement with the actual data, when the pseudo-second-order reaction rate equation was utilized for the computation. This observation indicates that the adsorption of MGon nano-bentonite and MgO-impregnated clay proceeds through a mechanism described by a second-order kinetics equation. According to a study conducted by Taher et al.^58^, the adsorption of the Congored dye onto acid-activated bentonite exhibits pseudo-second-order kinetics.

### Thermodynamic study

The thermodynamic adsorption qualities depend greatly on temperature. The effect of adsorption temperature on the MG adsorption of nano-bentonite and MgO-impregnated clay was investigated at various temperatures (298, 303, 308, 323, and 343 K). During the study on the thermodynamics of dye adsorption, 50 mg/L of dye and 1 g/L of two distinct adsorbents were used at temperatures of 25 °C, 30 °C, 35 °C, 50 °C, and 70 °C.The rate Eq. (15) and the van’t Hoff equation can be used to calculate the thermodynamic parameters, such as changes in the standard free energy (G), enthalpy (H), and entropy (S), related to the adsorption process (16). The rate equation is represented as follows^55,59^:

15 $${\mathrm{lnK}}_{\mathrm{L}}= \frac{{-\Delta \mathrm{H}}^{^\circ }}{\mathrm{RT}}+ \frac{{\Delta \mathrm{S}}^{^\circ }}{\mathrm{R}},$$

16 $$\Delta G^\circ = \Delta H^\circ -T\Delta S^\circ .$$

**Figure 16:**
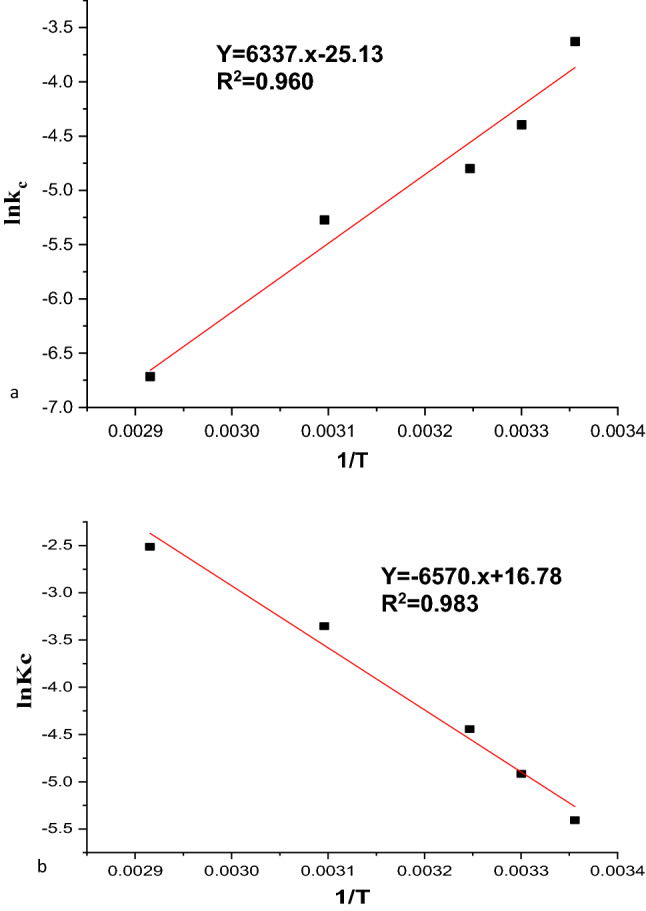
Thermodynamic for the adsorption of MG onto (**a**) nano bentonite and (**b**) MgO impregnated clay.

Here, ΔG^0^ is the free energy change of the sorption process (kJ/mol), and KC is the ratio of the equilibrium concentration of the MG ions on the adsorbent to the equilibrium concentration of the MG dye ions in the solution. R is the ideal gas constant (8.314 J/(mol K)),and T is the adsorption temperature in K. After plotting ΔG° against temperature, a linear relationship was achieved. The slope and intercept of the plot were used to compute the values of ΔS° and ΔH°. The results showed that ΔG° calculated from 25 to 70 °C were all negative, indicating that the adsorption of MG solution onto nano-bentonite and MgO-impregnated clay was feasible and spontaneous. Furthermore, analyses of the change inenthalpy (ΔH°) and entropy (ΔS°) were performed using the linear relationship (Eq. 12). Figure 16a,b shows the thermodynamic plot of ΔG° versus T to calculate ΔH° and ΔS°. Table 6 shows that the corresponding ΔH° and ΔS° values for Malachite green adsorption onto nano-bentonite obtained from the intercept and slope of the plot were equal to − 52.68 kJ/mol and − 0.2089 kJ/mol·K, respectively. The negative value of ΔH° in nano-bentonite showed that the adsorption was exothermic and indicated the possibility of chemisorption. These results are consistent with previous reports on the adsorption of MG on AC of rubber seeds, where MG adsorption was a chemisorption process^46^. The ΔH° and ΔS° values for Malachite green adsorption onto MgO-impregnated clay were 54.6 kJ/mol and 0.19 kJ/mol·K. Furthermore, a positive ΔH° value in MgO-impregnated clay adsorbent activity showed that adsorption was endothermic and suggested the possibility of physisorption. This result corresponds with the findings in the section on the temperature impact, which showed that adsorption capacity increases as temperature rises.

**Table 6:** Thermodynamic parameters of MG adsorption activity of nano bentonite and MgO impregnated clay.

Adsorbent	Temperature, K	DG, kJ/mol	DH, kJ/mol	DS, kJ/mol	R2
Nanobentonite	298	0.00957557	− 52.6858	− 0.20893	0.96
	303	0.01062022			
	308	0.01166487			
	323	0.01479884			
	343	0.01897745			
MgO impregnated nano bentonite	298	0.01304932	54.62298	0.13959	0.98
	303	0.01235178			
	308	0.01165423			
	323	0.0095616			
	343	0.00677142			

### Adsorption isotherm

Studies on the adsorption isotherms, such as the Freundlich, Langmuir, and Temkin isotherms, can be used to examine the effectiveness of the adsorbent material used for adsorption. Moreover, they can be used to determine the nature of the interaction between the adsorbed matter and the adsorbent^60,61^.

#### Langmuir adsorption isotherm

According to Ref.^15^, the Langmuir isotherm model was used to compute the maximal adsorption capacity resulting from complete monolayer coverage on the adsorbent surface and is shown as follows:

17 $$\frac{{C}_{e}}{{q}_{e}}= \frac{{C}_{e}}{{Q}_{max}} + \frac{1}{{Q}_{max}{K}_{L}}.$$

**Figure 17:**
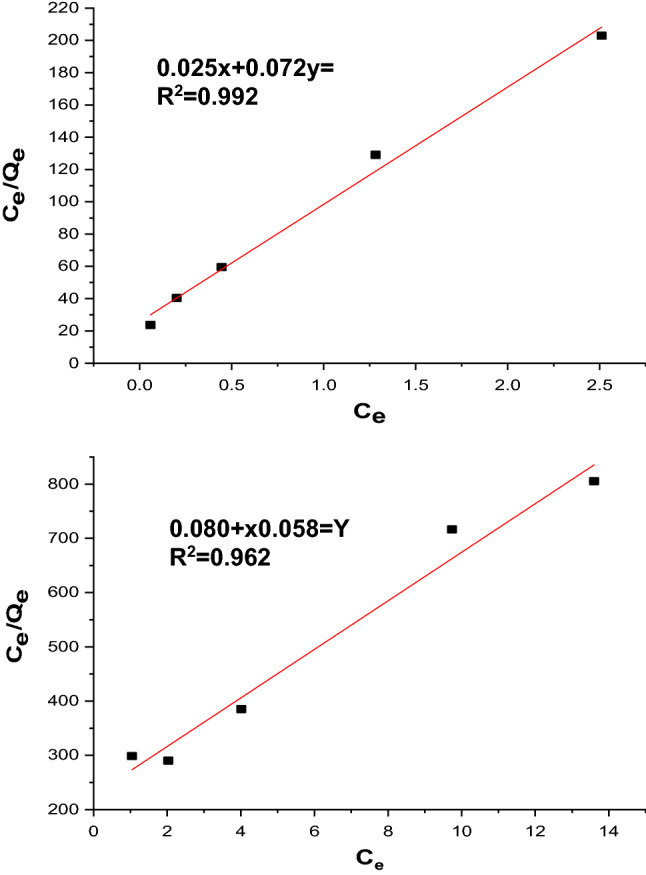
Langmuir (**a**) nano bentonite and (**b**) MgO impregnated clay plots for adsorption of MG.

Here, qm is the monolayer adsorption capacity (mg/g), qe is the equilibrium adsorption amount of the adsorbate, and Ce (mg/L) is the equilibrium adsorbate concentration. Regarding the adsorption rate (L/mg), KL is the Langmuir isotherm constant. By charting Ce/qe versus Ce, the values of qm and KL at various amounts of nano-bentonite and MgO-impregnated clay can be determined in the range of 0.99 and 1.2 L/mg Fig. 17a,b. A dimensionless constant called the separation factor RL may be used to express the essential properties of a Langmuir isotherm.

18 $$\mathrm{RL }= 1\left(1 +\mathrm{ KL Ce}\right).$$

Here, RL is the separation term, and Co is the initial concentration of the dye solution (mg/L). The effect of the isotherm shape on “favorable” or “unfavorable” absorptions was considered^62^. According to the RL values between (0–1), the isotherm is either unfavorable (RL > 1), linearly favorable (RL = 1), and or irreversible (RL = 0). Results from this experiment’s use of nano-bentonite and MgO-impregnated clay were observed for RL between 0.002 and 0.009, indicating that the adsorption was irreversible favorable. Table 7 shows the findings of MG removal on nano-bentonite and MgO-impregnated clay using the Langmuir model. The R^2^ in Table 7 showed strong positive proof of the adsorption of MG ion adsorbents following the Langmuir isotherm. The suitability of the linear form of the Langmuir model to nano-bentonite was confirmed through the high correlation coefficients R^2^ > 0.992.Conversely, the linear form of the Langmuir model to MgO-impregnated clay was slightly fit with the regression coefficients (R^2^) value (0.962%). This shows that the Langmuir isotherm can provide a decent sorption model. Moreover, the adsorption capacities of the nano-bentonite and MgO-impregnated clays were 13.8 and 17.2 mg/g, respectively. This result corresponds with^6^, who discovered that the adsorption capacity of CuFe_2_O_4_ for MG is 22 mg/g.

**Table 7:** Equilibrium isotherm modeling of MG adsorption onto both adosorbents nano bentonite and MgO impregnated clay.

Isotherm	Nano bentonite	MgO-clay
Langmuir		
qe (mg/g)	13.8	17.2
R2	0.992	0.962
R_L_	0.002	0.009
Freundlich		
N	2.3	1.3
KF (L/mg)	109.5	3.6
R2	0.973	0.982
Tempkin		
B (J/mol)	0.005	0.004
A _T_ (L/mg)	4.055	2.11
R2	0.965	0.982

#### Freundlich adsorption isotherm

According to the Freundlich isotherm model, adsorption occurs on a heterogeneous surface with a non-uniform heat distribution over the adsorbent surface. The linearized form of the Freundlich model is given as follows^63^:

19 $$ln{q}_{e}=ln{K}_{F}+\frac{1}{n}ln{C}_{e}.$$

**Figure 18:**
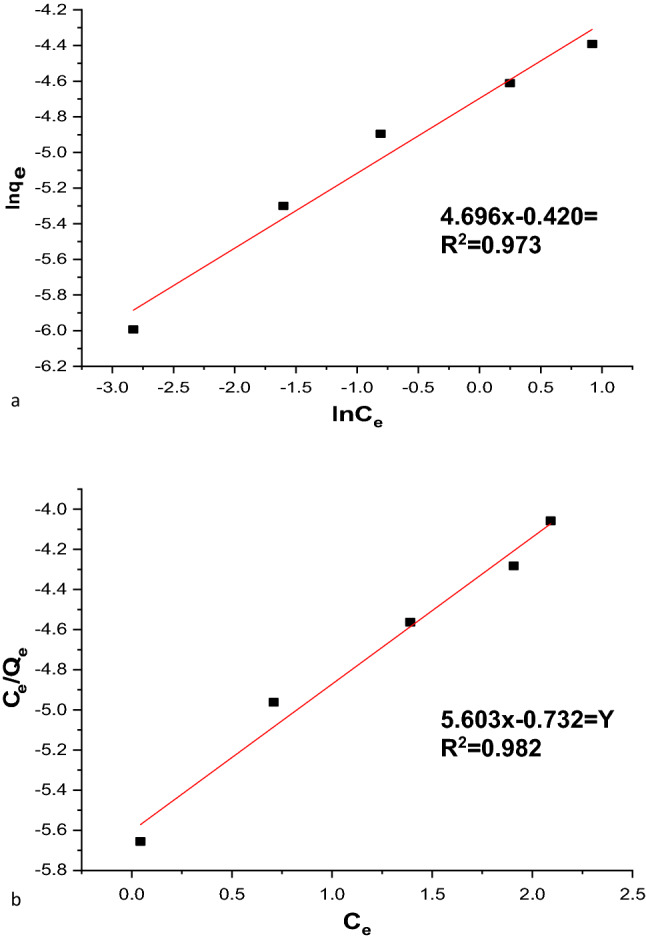
Freundlich (**a**) nano bentonite and (**b**) MgO impregnated clay plots for adsorption of MG.

Here, qe is the amount adsorbed per unit mass of the adsorbent, Ce is the equilibrium concentration, and 1/n and Kf are the Frendulich constants. The values of *1/n* represent the nonlinearity of the relationship between adsorption and solution concentration. Adsorption is linear if n equals unity, chemical adsorption is implied if the value of n is below unity, and advantageous physical adsorption is implied if n is above unity. Figure 18a,b shows the plots of lnqe versus lnCe for the adsorption of MG dye on nano-bentonite and MgO-impregnated clay. The values of Kf (mg/g) and n are obtained from the intercept and slope, respectively. The values were (1.9 mg/g) and (2.3) for nano-bentonite and (3.6 mg/g) and (1.3) for MgO-impregnated clay. The R^2^ values for nano-bentonite and MgO-impregnated clay are approximately 0.973% and 0.982%, respectively. This indicates that both systems are favorable and that the MgO-impregnated clay has a higher adsorption capacity. Therefore, the value of 1/n showed the applicability of the adsorbent used over the range of dye solution concentrations that significantly affect the Freundlich adsorption isotherm. The values of n for the MG dye adsorption on nano-bentonite and MgO-impregnated clay were 2.3 g/L and 1.6 g/L, respectively, indicating that the adsorption occurred as a chemical process for n > 1.

#### Tempkin isotherm

The adsorption energy changes and the surface of the adsorbent toward the adsorption of different species in diverse mixes were assessed using the Tempkin adsorption isotherm. The R^2^ value and decreased error analysis were effective and efficient criteria. The model has typically been used in the following format (Eq. 20):

20 $${q}_{e}=\frac{RT}{b}\mathit{ln}\left({K}_{T}\right)+\frac{RT}{b}ln\left({C}_{e}\right).$$

**Figure 19:**
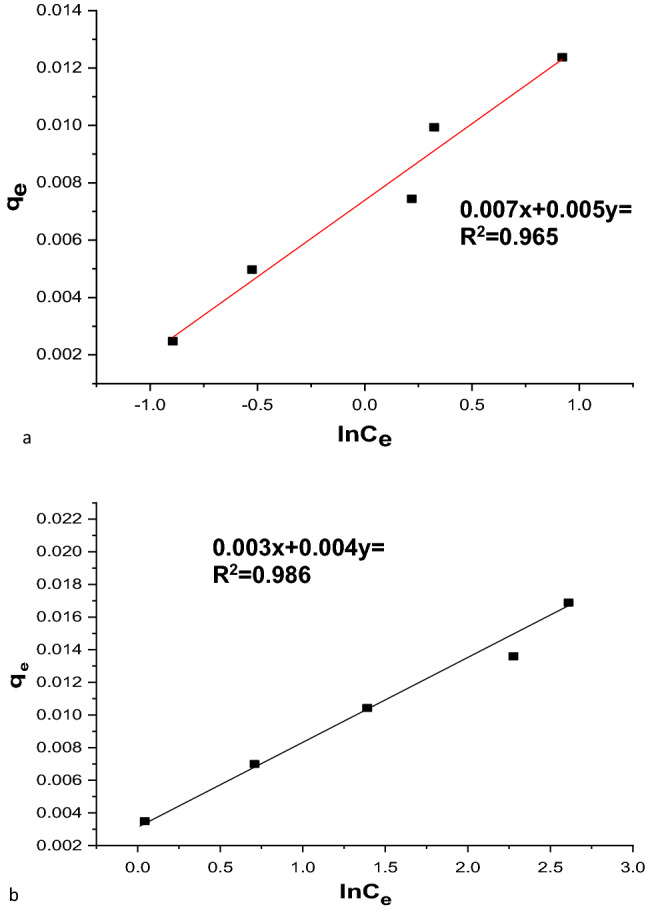
Temkin (**a**) nano bentonite and (**b**) MgO impregnated clay plots for adsorption of MG.

Here, β = (RT)/b,Tis the absolute temperature in Kelvin, and R is the universal gas constant(8.314 J/(mol K)); the constant β correlates with the heat of adsorption^60^. As shown in Table 7 and Fig. 19a,b, applying the experimental equilibrium data to Eq. (20) demonstrated excellent and reasonable applicability of the model in explaining and interpreting MG adsorption on nano-bentonite and MgO-impregnated clay. In the Temkin isotherm, positive AT values of 1.4 L/mg and 2.1 L/mg for nano-bentonite and MgO-impregnated clay, respectively, showed that the process was endothermic. The Temkin model also showed a high R^2^ value, indicating a chemisorption process rather than a physisorption one. The results obtained correspond with those reported by Gündüz^60^. Furthermore, the R^2^ values realized using the Tempkin model were similar to those observed using the Langmuir and Freundlich equations.

### Comparison of the adsorption capacities of nano-bentonite and MgO-impregnated clay with various adsorbents

In Table 8, the maximum adsorption capacities (q_max_) of nano-bentonite and MgO-impregnated clay for MG adsorption are compared to the qmax of other adsorbents in the literature. The maximum adsorption capacities of MgO-impregnated clay and nano-bentonite for MG are shown to be greater than those of other adsorbent materials, which may be attributed to the strong adsorption capacity of nano-bentonite. Additionally, because the adsorbent surface was negatively charged during the experiment, the electrostatic attraction between positively charged adsorbate species and adsorbent particles grew, which caused additional MG to be adsorbed.

**Table 8:** The experimental range and levels of input process variables assessed.

Variables	Code	Levels		
		− 1	0	1
pH	A	5	7	9
Dye concentration	B	5	50	100
Temperature (C)	C	30	35	40
Contact time	D	24	48	72

### Parameters for the performance and optimization of MG adsorption

Analyzing the interaction between working factors using conventional methods is challenging. Consequently, predictions of operational factors and their synergistic impact are frequently based on assumptions. Operational factors can be simultaneously and efficiently analyzed, and the degree of interaction can be assessed using RSM. Due to the operational factor screening, the ideal conditions for MG adsorption were determined. The optimum condition of processing factors for MG adsorption was obtained at pH 9,with an initial concentration of 50 mg/L and a 4.0 g/L adsorbent dosage within 35 °C for nano-bentonite. Compared with MgO-impregnated clay, the optimum condition of processing factors was obtained at pH 9, with an initial concentration of 50 mg/L anda4.0 g/L adsorbent dosage within 40 °C. Under these circumstances, the high decolorization efficiency was 97.53% and 93.9% for nano-bentonite and MgO-impregnated clay, respectively.

### Optimization of Malachite green decolorization using statistical design

**Figure 20:**
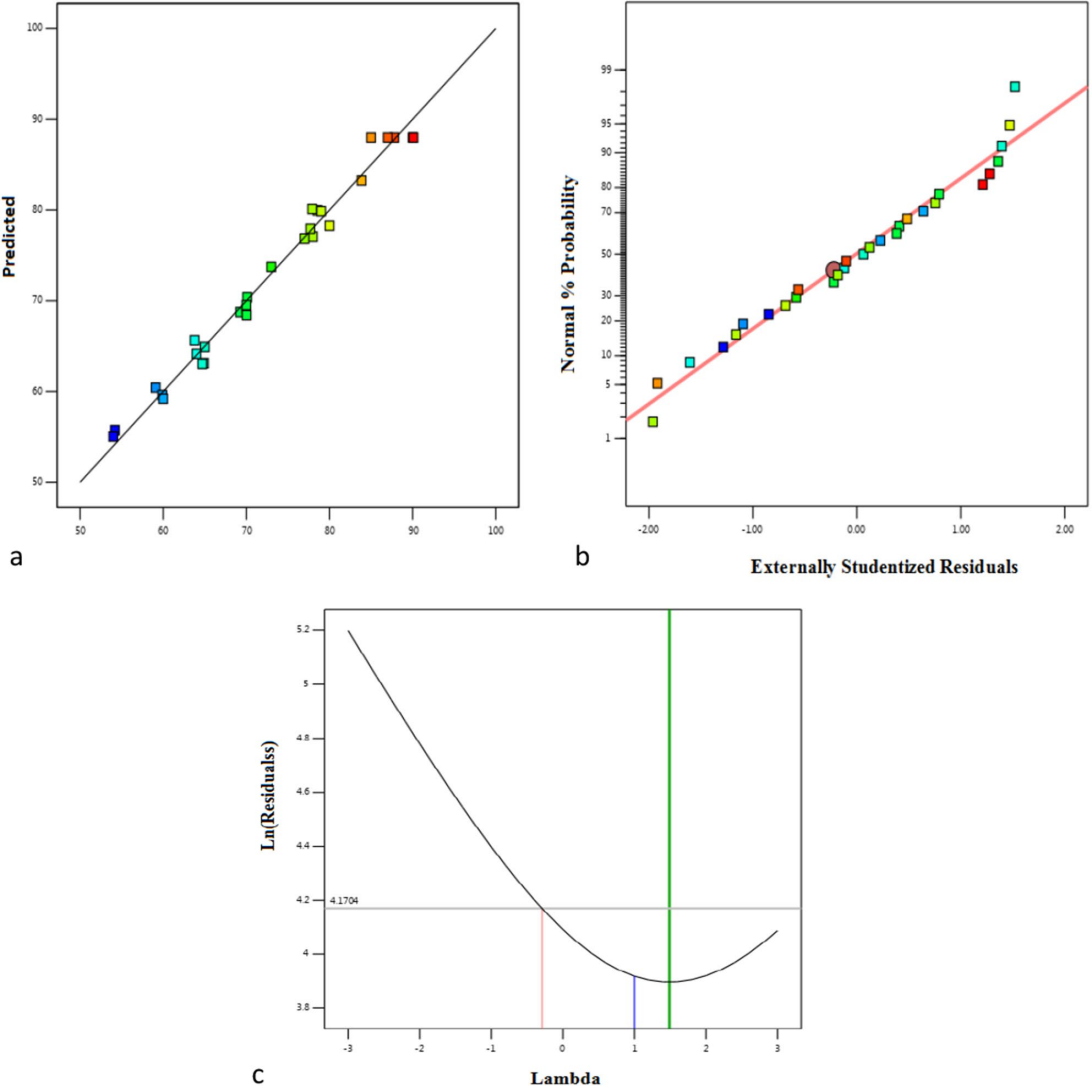
(**a**) The actual and predicted values for the decolourization of MG dye by immobilized *Mucor* sp., (**b**) normal % probability and (**c**) the Box–Cox plot.

Supplementary Table 1 shows that the Box–Behnken design with four variables (pH, initial concentration, contact time, and temperature) was used to improve the decolorization process. Supplementary Table 2 shows the experimental and predicted values of the percentage decolorization. The second-order response surface polynomial function allowed the prediction of ideal dye operating conditions. Figure 20a,b shows that the experimental response values for MG decolorization correspond with the predicted response values, the normal probability, and the studentized residual plot.

21 $$\begin{aligned} & {\text{Y}} = 87.98 + 2.09167 \times {\text{A}} + - 0.153333 \times {\text{B}} + - 0.0158333 \times {\text{C}} + 1.53917 \times {\text{D}} + - 2.5 \times {\text{AB}} \\ & \quad + - 7.25 \times {\text{AC}} + 1.575 \times {\text{AD}} + 4.61 \times {\text{BC}} + 7.265 \times {\text{BD}} + 0.0925 \times {\text{CD}} + - 18.4612 \\ & \quad \times {\text{A}}^{2} + - 9.34375 \times {\text{B}}^{2} + - 5.145 \times {\text{C}}^{2} + - 4.3425 \times {\text{D}}^{2} \\ \end{aligned}$$

According to Table 9, the ANOVA results for the quadratic regression model indicated that the model was significant. The superior F value (60.99) and reduced *P* values (< 0.0500) of Malachite green show that the model terms were significant. The variables A, D, AB, AC, BD, BD, A2, B2, C2, and D2 were determined to be significant model terms for decolorization based on the P values. Furthermore, according to the results of the ANOVA in Supplementary Table 2, the linear effects of the dye temperature, pH, and concentration were found to be increasingly important for MG dye decolorization. According to the “lack of fit F value of 0.715,”the lack of fit was insignificant regarding the pure error. An insignificant lack of fit was regarded as a reliable indicator that the model would be good. The fit of the model was also expressed by the coefficient of regression R^2^.The predicted R^2^ of immobilized *Mucor* sp.was 0.9837, which is consistent with the adjusted R^2^ of 0.967. These findings indicate that the developed model was satisfactory and that the values of the independent factors were accurate with minimal error. The range of the projected response regarding the associated error was measured with adequate precision. A ratio of at least 4 is acceptable; however, a ratio higher than 4 is preferable. The ratio of 24.1 for *Mucor* sp. was high, indicating the reliability of the experimental data. Moreover, according to Supplementary Table 2, the coefficient of variation (CV%) values for *Mucor* sp. obtained in the study are relatively small, with 2.6. This indicated that the deviations between the experimental and predicted values were low. Figure 20c shows a graph of the Box-Cox diagram of model changes in MG removal (%) using *Mucor* sp. composite determined by a quadratic polynomial. The best lambda value (λ = 1.49) is between the two red vertical lines, so no data transformation is required. The red line shows the minimum (− 0.2900) and maximum (3.32) values, as well as lambdas at 95% confidence interval value.

**Table 9:** Various kinetic models for effect of substrate on growth rate.

Model	Equation	μmax (h^–1^)	Ks (mg/L)	Ki	Sm	n	m	RMSE	AICc	AF	R2
Haldane	$\upmu =\frac{\mu maxS}{(S+KS+\frac{S^2}{Ki})}$	1.02	70	70	–	–	–	0.05	− 31.8	0.94	0.961
Han and Levenspiel	$\upmu =\frac{\mu maxS\left[1-\left(\frac{S}{Sm}\right)\right]n}{\left(S+KS-\right)\left[1-\left(\frac{S}{Sm}\right)\right]m}$	1.1	100		360	1	1	0.06	− 26.8	0.91	0.922
Luong	$\upmu =\frac{\mu maxS\left[1-\left(\frac{S}{Sm}\right)\right]n}{(S+KS)}$	1.01	140		400	1		0.09	− 20.2	0.88	0.843
Aiba	$\upmu =\frac{\mathrm{\mu maxS exp}(-\mathrm{ S}/\mathrm{Ki})}{ (\mathrm{S }+\mathrm{ KS})}$	1.6	150	150	–	–	–	0.06	− 27.45	0.91	0.822
Monod	$\mu max=\frac{S}{KS}$	0.9	120		–	–	–	0.3	7.5	0.79	0.69

#### Interactive impact of pH on MG dye decolorization

The biosorption of Malachite green by the fungus was investigated for a pH range of 5–9.

**Figure 21:**
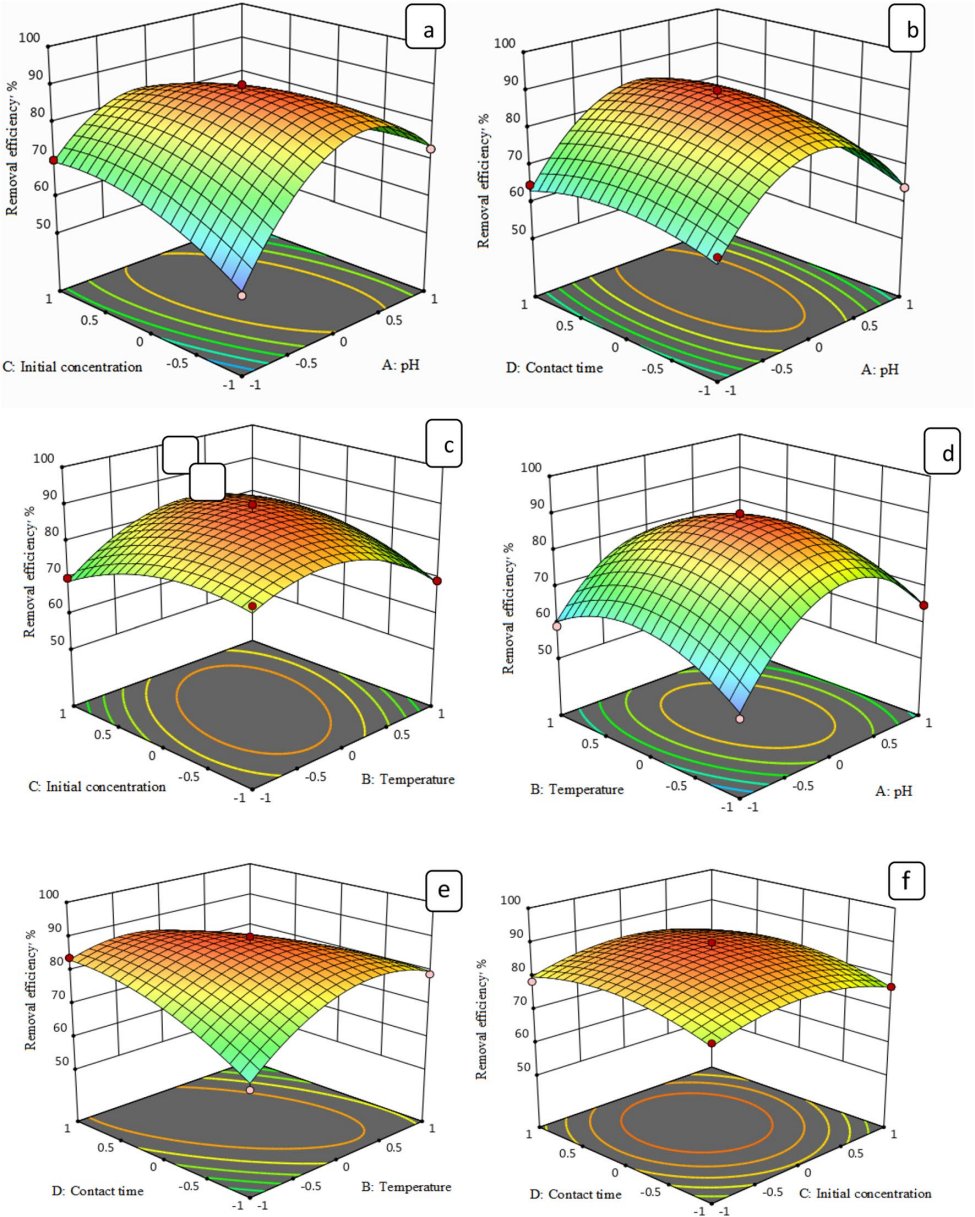
(**a**) the cumulative impact of pH and contact time, (**b**) the cumulative impact of pH and MG dye concentration, (**c**) the cumulative impact of pH and temperature, (**d**) the cumulative impact of contact time and MG dye concentration, (**e**) the cumulative impact of contact time and temperature, (**f**) the cumulative impact of temperature and MG dye concentration.

The maximum degree of decolorization (97.8%) was reached at a pH of 7.0, while at a pH of 9, the decolorization rate decreased to 40%. Figure 21a,b shows that the efficacy of dye decolorization using immobilized *Mucor* sp. decreased with rising pH levels. Moreover, Fig. 21c shows that the removal efficiency was 54% at a pH of 5.0 and 30 °C, and it improved to 87.8% at a pH of 7 and 30 °C. Similar findings were made by Ref.^64^, who discovered that the efficiency of decolorization of Malachite green using *Aspergillus niger* was about 97% at a pH of 7.

#### Interactive impact of temperature on MG dye decolorization

Various environmental factors influenced the degradation of Malachite green using *Mucor* sp. This fungal strain degraded Malachite green effectively from 298 to 303 °C (Fig. 21c,e,f). Figure 21c,e,f shows that the rate of Malachite green decolorization increases as the temperature rises from 25 to 30 °C. Furthermore, Malachite green decolorization by *Mucor* sp. ON934589.1 reached a maximum of 91.54% at 303 °C. Conversely, as the temperature rose to 313 °C, the decolorization activity decreased (53%) due to the loss of cell viability or inactivation of the decolorizing enzymes^65^. Arunprasath et al.^4^ observed that the optimum temperature (30 °C) was the optimum temperature for the decolorization (92%) of Malachite green dye by *Lasiodiplodia* strains.

#### Interactive impact of concentration on MG dye decolorization

The adsorption behavior of MG was studied in concentrations of 5–200 mg/L at a pH of 7.0. In addition, 87.7–97.4% of Malachite green was eliminated by the immobilized fungus at 5–100 mg/L. The decolorization efficiency of *Mucor* sp*. *(ON934589.1) fell below 64% when the starting concentration of Malachite green approached 150 mg/L. These findings suggest that high concentrations of Malachite green impede the development of *Mucor* sp. (ON934589.1). Figure 21b shows the impact of the initial concentration and contact time on the removal of Malachite green dye using immobilized fungus. The immobilized fungus was suppressed at 150 mg/L of Malachite green due to the existence of sulfonic acid on the aromatic ring formed in the medium by the increased concentration of Malachite green, which inhibited the nucleic acid synthesis and microbial cell proliferation^66^. The findings of this investigation corresponded with those of Ref.^67^, who found that *Aspergillus fumigates* immobilized in polyurethane foam had an optimum Malachite green decolorization percentage of about 97.52% (40 mg/L),which reduced to 23% at 70 mg/L.

#### Impact of contact time on MG dye decolorization

At the optimum dye concentration (50 mg/L) and biosorbent dosage (6 g/L), the impact of contact time on adsorption was examined from 24 to 72 h. Figure 21a,d shows the influence of contact time on the elimination of the MG dye. The range of the MG’s adsorption efficiency was 18 to 72 h, corresponding to 72% and 97%, respectively. Based on the data, 40 h was determined to be the equilibrium time in the sorption process because no further improvement was observed after reaching maximum adsorption. The high removal efficiency at the beginning of the contact time of 40 h was due to the large surface area available for dye adsorption during the initial stage, and the adsorbent’s capacity gradually depleted over time, as the few remaining vacant surface sites became tough to occupy because of repulsive forces between the solute molecules on the solid and bulk phases^68,69^. Our results correspond with those of Ref.^70^, who observed that *Lasiodiplodia sp*. could decolorize 81% of Malachite green within 36 h.

### Biodegradation kinetics: theoretical considerations

The Monod model is used to represent the relationship between the limiting substrate concentration and the specific growth rate using Eqs. (19) and (20).

22 $$\mu =\frac{\mu maxS}{S+KS},$$

23 $$\mu =\frac{1}{x}\frac{dx}{dt}.$$

Here, µ, µmax, and Ks were determined as the biodegradation experiment data. The magnitude of Yx/s was estimated from the slope of the graph of dX dt versus S. The original Monod model becomes inadequate when a substrate prevents biodegradation. Monod derivatives with substrate inhibition adjustment Eqs. (24 and 32), suggested by the Haldane, Aiba–Edward, Luong, Han, and Levenspiel models, have been used to assess the impacts of inhibition at a high substrate concentration and stimulation at a low concentration of substrate^71–74^. Here, S and μ are the substrate concentration and the specific growth rate, respectively; μmax is the maximum specific growth rate; n and m are experimental constants; Ks is the half substrate saturation coefficient, and Sm is the critical inhibitor concentration (mg/L) above which growth ceases.

24 $$q=\frac{1}{X}\frac{ds}{dt},$$

25 $$q=\frac{QmaxS}{K2+S},$$

26 $$\frac{dy}{dx}=-\frac{\mathrm{qmaxSX}}{\mathrm{Ks}+\mathrm{S}},$$

27 $$\frac{dy}{dx}=-Yx/s\frac{ds}{dt},$$

28 $$\upmu =Yx/sQ,$$

29 $$\mu = \frac{umaxS}{{S + Ks + [1 - \left\lfloor {\frac{s2}{{K1}}} \right\rfloor m}},$$

30 $$\upmu =\frac{\mu maxS\left[1-\left(\frac{S}{Sm}\right)\right]n}{(S+KS)},$$

31 $$\mu = \frac{{umaxS\left\lfloor {1 - \frac{{\text{s}}}{{{\text{Sm}}}}} \right\rfloor {\text{n}}}}{{S + Ks[1 - \left\lfloor \frac{s}{Sm} \right\rfloor m}},$$

32 $$\upmu =\frac{\mathrm{\mu maxS \, exp}(-\mathrm{ S}/\mathrm{Ki})}{ (\mathrm{S }+\mathrm{ KS})}.$$

Here, µ = specific growth rate of biomass, µ_max_ = maximum consumption rate constant, S = substrate concentration, K1 = the substrate inhibition constant (mg/L), Ks = Monod constant, and Sm = decisive inhibitor concentration (mg/L); n and m are experimental constants.

#### Biodegradation kinetics of Malachite green

For the range of concentrations in the study (5–200 mg/L), the length of the lag phase t_0_ grew exponentially with the Malachite green concentration Supplementary Fig. 1a. Thus, Malachite green was considered to have an inhibiting effect on microbial development at high concentrations. These findings correspond with those previously recorded for mixed cultures^36^. We compared the evolution of the lag phase to the particular growth rate to gain advanced insight into the impact of the lag phase. Two trends were observed, one below Supplementary Fig. 1a,c and one above Supplementary Fig. 1b the 100 mg/L Malachite green concentration. The time of the lag phase t_0_ rose linearly with an increase in the maximum specific growth rate when the concentration of Malachite green Supplementary Fig. 1a was less than 100 mg/L. However, a contrary trend was observed for concentrations greater than 100 mg/L supplementary Fig. 1b, where the length of the lag phase t_0_ increased as the maximum specific growth rate decreased. The findings of the curve fitting Supplementary Fig. 2 using models such as Monod, Luong, Aiba–Edward, Han, and Levenspiel did not match the experimental results and were excluded. The Luong model provided reasonably acceptable results according to software output and visual examination. The accuracy and statistical analyses of the four kinetic models used in the study revealed that Haldane was the most accurate model, having the minimum root-mean-square error and AICc values and the maximum adjusted R^2^. Table 10 shows the Af and Bf values. The Af and Bf values for Haldane were significant and closest to 1.0.The results of an F-test indicated that the Haldane model was better than the Aiba–Edward, Han, Levenspiel, and Luongmodels, which were 96.1%,92.2%, 84.43%, and 82.2%, respectively. These results indicate that the Haldane model was superior to the rest. The computed values for the Haldane constants in this work, such as the inhibition constant rate symbolized by the maximal growth rate and half-saturation constant umax, Ks, and K_i_, were 1.02 h^−1^, 70 mg/L, and 70 mg/L.

**Table 10:** Adsorption capacities of different adsorbents previously reported for the removal of MG compared with nano bentonite and MgO impregnated clay.

Adsorbents	Adsorption capacity (mg/g)	References
Shells seeds of Ziziphus spina christi	48.7	^17^
Nano bentonite	13.8	Present study
MgO impregnated clay	17.2	Present study
Prosopis cineraria saw dust treated by Sulphuric acid	65.8	^75^
NaOH- modified breadnut peel	352.2	^46^
Alg-Fe_3_O_4_ nanoparticles	47.8	^76^
Coal-associated soil	89.9	^77^
Sea shell powder	42.3	^78^
Organically modified hydroxyapatite	188.5	^79^
Calcium alginate nanoparticles	277.7	^80^

### Mechanism of MG removal

The fungi decolorize MG via biosorption and biodegradation as their main mechanisms. Biosorption occurs immediately as dye molecules bind to the functional groups present on the surface of fungal mycelium, reflected by the rapid decolorization rate, which plateaus once the binding sites are saturated and equilibrium is achieved^81^. Biodegradation involves enzymatic breakdown of the dyes into smaller molecules. Enzymatic degradation requires a close fit of the polymer chain into the enzyme active site conformational flexibility is necessary for high degradability^23^. Immobilization of fungal cells may stably maintain the production of various enzymes at levels higher than those achieved with suspended or pellet forms. Moreover, the immobilization of fungal biomass increases fungal resistance to environmental stresses, such as the presence of toxic molecules at high concentrations.Immobilization improves decolorization efficiency of biomass due to less dense fiber packing in comparison with the free fungal biomass. This is because the fungi has a larger surface area available for dye adsorption. The increase in the surface area of fungal biomass tends to reduce the mass transfer limitations, which in turn increases access to pollutant degradation. Immobilization may allow the use of the system repeatedly, allowing easier liquid–solid separation and avoiding clogging phenomena^82^. The present study has revealed that *Mucor* sp. composite was able to decolorize 50 mg/L malachite green (87.8%) within 72 h at pH 7.5 and temperature 30 °C were optimized for dye degradation. The most widely researched are white rot fungi, such as *Phanerochaete chrysosporium*, *Bjerkandera* sp.,* Trametes versicolor*, *Irpex lacteus*, and *Pleurotus ostreatus*, which produce enzymes, such as lignin peroxidase, manganese peroxidase and laccase. They can degrade many aromatic compounds due to their non-specific enzymatic activity^83^. Reference^84^ observed that MG dye degradation occurred for 6 days by two fungal strains *Aspergillus flavus* (99.78%) and *Alternaria solani* (91.72%), which was up to 10.95 mg/L, when MG was a sole source of carbon. But degradation had increased up 97.43 and 96.91 at 18.25 mg/L%, respectively, when an extra carbon source was added to the medium. As described previously, white-rot fungi are capable of decolorizing dyes significantly,and in most cases, this is due to the activities of lignin peroxidase (LiP)22 and Mn-dependent peroxidase (MnP). Some studies have demonstrated laccase (Lac)-mediated dye decolorization^85^. Reference^63^ who reported that the immobilized laccase onto TiO_2_–ZrO_2_–SiO_2_– material degraded Alizarin Red S (ARS), Remazol Brilliant Blue R (RBBR), and Reactive Black 5 (RB5)from an aqueous solution at a concentration 5 mg/L under optimal process conditions, which were pH 5 and 25 °C, with degradation efficiency reached 100%, 91%, and 77%, respectively. In absorption processes, activated carbon is a highly effective and versatile material. MG dye was reported by Ref.^86^ who showed that they produce an extracellular oxidoreductive, nonspecific, and non-stereo selective enzyme system including lignin peroxidase, tyrosinase, manganese peroxidase, and laccase to destroy MG dye.

### Microbial toxicity

The results of the microbial toxicity study demonstrated that the medium containing 100 mg/L (control) MG had inhibition zones, indicating the toxicity of MG to the *E. coli* and *Pseudomonas aeruginose* and *Staphylococcus aureus* strains. The treated sample did not demonstrate any growth inhibition when compared to the untreated 100 mg/L MG, showing that the formation of the adsorption process was nontoxic Supplementary Fig. 3. This suggests that the effluent might not have any negative effects on its surroundings when released into water bodies.

### Reusability of the various adsorbents

The recyclability of nano-bentonite and MgO-impregnated clay adsorbents for MG removal was studied. Supplementary Fig. 4 shows the results of recycling studies, and the graph shows that there was a minimal loss in MG removal up to seven cycles. However, after seven cycles, the MG removal effectiveness of the nano-bentonite and MgO-impregnated clay declined from 93 to 86.85% and 92.2 to 83%, respectively. Reference^68^ reported that the reusability of a *Curcuma caesia* based on the AC adsorbent for the removal of Malachite green was sustained, even after eight cycles at 81%.

## Conclusion

This study has shown that nano-bentonite, MgO-impregnated clay, and immobilized *Mucor* sp. ON934589.1are effective adsorbents for removing MG from aqueous solution. The RSM based on BBD combining was utilized to investigate the effect of four different process variables (adsorbent dosage, initial MG concentration, pH, and contact time) on the dye removal efficiency of nano-bentonite, MgO-impregnated clay, and immobilized *Mucor* sp. ON934589.1in aqueous solution. The relative effects of the interactions of the mentioned process variables were also successfully analyzed. The corresponding experimental values of dye adsorption were found to be 0.986%, 0.973%, and 0.983%, which extensively corresponded to the optimal values(0.93%, 0.91%, and0.87.8%) predicted by the model RSM for nano-bentonite, MgO-impregnated clay, and *Mucor* sp. (ON934589.1), respectively. The optimal Malachite green removal efficiency of MgO-impregnated clay was found at a pH of 9.0, an initial MG concentration of 50 ppm, a dosage of 0.7 g, and a contact time of 60 min. However, the Malachite green removal efficiency of nano-clay was observed to be optimal at 35 °C, 7.0, 60 min, 1 g/L, and 50 mg/L. The Malachite green adsorption isotherm on MgO-impregnated clay showed maximum consistency with the Freundlich isotherm model, withan R^2^ value of 0.982. However, the Langmuir adsorption isotherm was better suited for nano-bentonite (R^2^ = 0.992). The adsorption activities of nano-bentonite and MgO-impregnated clay were matched to a pseudo-second-order model equation with R^2^ values of 0.996 and 0.995, respectively. Furthermore, nano-bentonite and MgO-impregnated clay were matched to the Temkin isotherm with R^2^ values of 0.965 and 0.986, respectively. The Gibbs free energy was positive for nano-clay (0.72–7.5 kJ mol) and negative for MgO-impregnated clay (− 4.07 to − 12.9). Moreover, nano-bentonite and MgO-impregnated clay showed enthalpy changes of − 0.151and 0.196, respectively. A high biodegradation efficiency of 87.8% was obtained during a 72 h decolorization examination of a dye using the isolated fungus *Mucor* sp. (GenBank accession no. ON934589.1).

## Supplementary Information

Supplementary Figure S1.

Supplementary Figure S2.

Supplementary Figure S3.

Supplementary Figure S4.

Supplementary Table 1.

Supplementary Table 2.

## Data Availability

All data generated or analyzed during this study are of our own work and it is our pleasure to be available publically. Connect with aurthor-mohamed_taha@nwrc.gov.eg.
