# Supplementary material for: Preparation and characterization of low-cost adsorbents for the efficient removal of malachite green using response surface modeling and reusability studies
Source: Sci Rep. 2023 Mar 18;13:4493. doi: 10.1038/s41598-023-31391-4 (PMC10024755; doi:10.1038/s41598-023-31391-4)
Supplement: Supplementary file 1 — Supplementary Figure S1. [file 41598_2023_31391_MOESM1_ESM.docx]

^
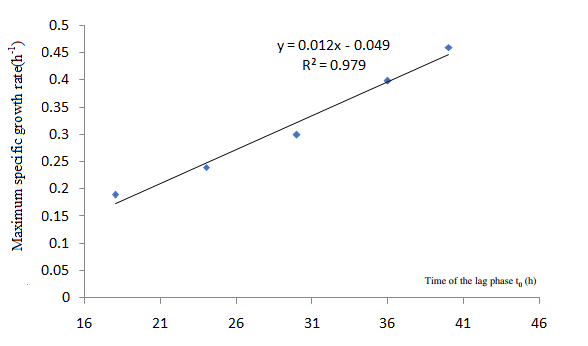

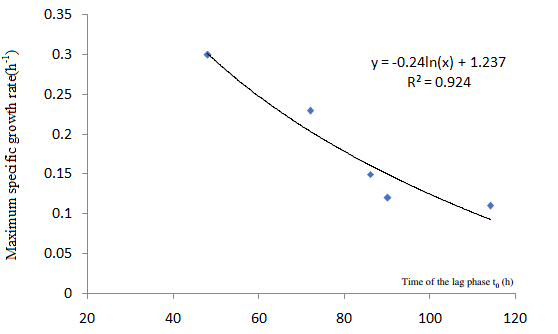

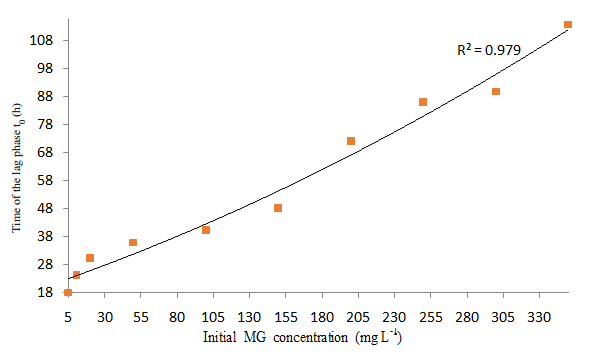
^

b

a

c

Fig. S1 Time of the lag phase (t0) versus the initial MG concentration (a), and (S_0_) Maximum specific growth rate μm versus the time of the lag phase t_0_ for S_0_ below (b), above (c) MG concentration mg L^-1^
