# Supplementary material for: Preparation and characterization of low-cost adsorbents for the efficient removal of malachite green using response surface modeling and reusability studies
Source: Sci Rep. 2023 Mar 18;13:4493. doi: 10.1038/s41598-023-31391-4 (PMC10024755; doi:10.1038/s41598-023-31391-4)
Supplement: Supplementary file 2 — Supplementary Figure S2. [file 41598_2023_31391_MOESM2_ESM.docx]

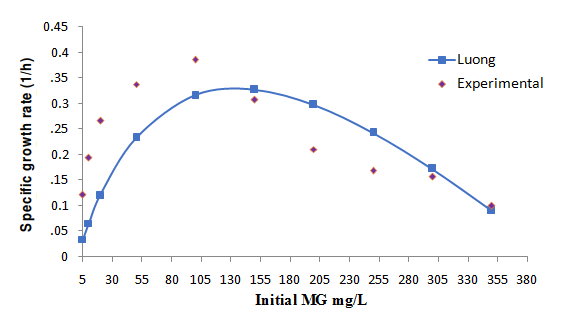

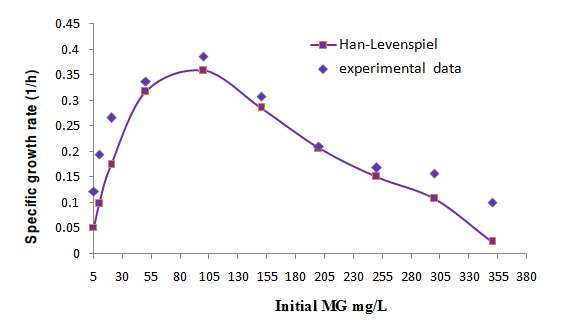

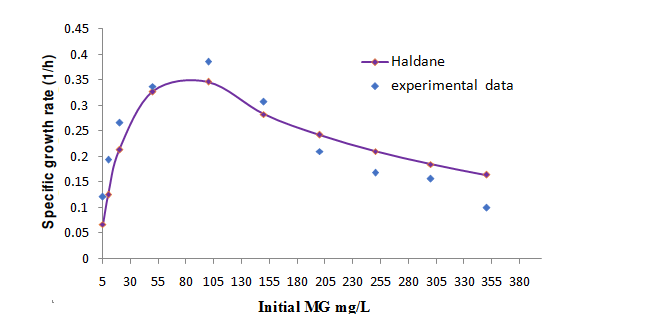

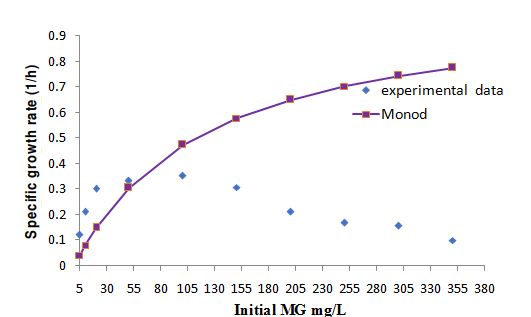

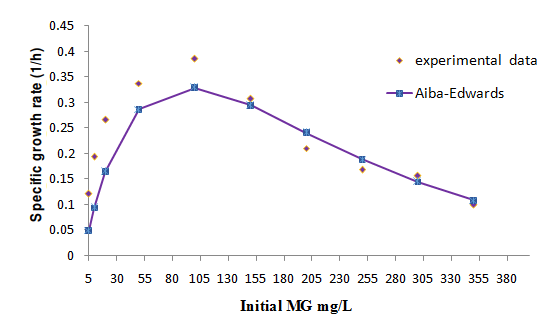


Fig.2 Experimental and predicted speciﬁc substrate consumption rates at diﬀerent MG concentrations due to diﬀerent models.
