# Supplementary material for: Preparation and characterization of low-cost adsorbents for the efficient removal of malachite green using response surface modeling and reusability studies
Source: Sci Rep. 2023 Mar 18;13:4493. doi: 10.1038/s41598-023-31391-4 (PMC10024755; doi:10.1038/s41598-023-31391-4)
Supplement: Supplementary file 4 — Supplementary Figure S4. [file 41598_2023_31391_MOESM4_ESM.docx]

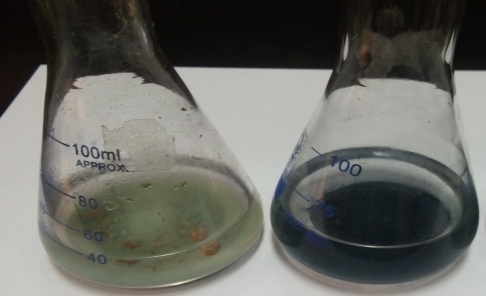


Fig. 4. Percentage removal (%) of the adsorption–desorption cycles of nano bentonite and (b) MgO impregnated clay
