# Supplementary material for: Preparation and characterization of low-cost adsorbents for the efficient removal of malachite green using response surface modeling and reusability studies
Source: Sci Rep. 2023 Mar 18;13:4493. doi: 10.1038/s41598-023-31391-4 (PMC10024755; doi:10.1038/s41598-023-31391-4)
Supplement: Supplementary file 5 — Supplementary Table 1. [file 41598_2023_31391_MOESM5_ESM.docx]

| Run | A:ph | B:tp | C:con | D:dose | Experimental | Predicted |
| --- | --- | --- | --- | --- | --- | --- |
| 1 | 1 | 0 | 0 | 1 | 70.1 | 70.38 |
| 2 | 0 | 0 | -1 | -1 | 78 | 77.06 |
| 3 | 0 | 0 | 0 | 0 | 90 | 87.98 |
| 4 | 1 | 0 | 1 | 0 | 60 | 59.20 |
| 5 | 1 | 0 | -1 | 0 | 73 | 73.73 |
| 6 | 1 | 0 | 0 | -1 | 64 | 64.15 |
| 7 | 0 | 1 | -1 | 0 | 69.26 | 68.74 |
| 8 | 0 | 0 | 1 | -1 | 77 | 76.85 |
| 9 | 0 | 0 | 0 | 0 | 90.1 | 87.98 |
| 10 | -1 | 1 | 0 | 0 | 59.1 | 60.43 |
| 11 | 0 | 1 | 0 | 1 | 70.03 | 68.41 |
| 12 | 0 | 1 | 1 | 0 | 77.7 | 77.93 |
| 13 | 1 | -1 | 0 | 0 | 65 | 64.92 |
| 14 | -1 | 0 | 1 | 0 | 70 | 69.52 |
| 15 | -1 | 0 | 0 | 1 | 64.7 | 63.05 |
| 16 | -1 | -1 | 0 | 0 | 54.2 | 55.74 |
| 17 | 0 | -1 | 0 | -1 | 63.78 | 65.64 |
| 18 | 0 | 1 | 0 | -1 | 79.01 | 79.87 |
| 19 | 1 | 1 | 0 | 0 | 59.9 | 59.61 |
| 20 | -1 | 0 | 0 | -1 | 64.9 | 63.12 |
| 21 | -1 | 0 | -1 | 0 | 54 | 55.05 |
| 22 | 0 | 0 | 1 | 1 | 77.92 | 80.11 |
| 23 | 0 | 0 | 0 | 0 | 87 | 87.98 |
| 24 | 0 | 0 | -1 | 1 | 78.55 | 79.96 |
| 25 | 0 | -1 | 0 | 1 | 83.86 | 83.25 |
| 26 | 0 | -1 | -1 | 0 | 80 | 78.27 |
| 27 | 0 | 0 | 0 | 0 | 87.8 | 87.98 |
| 28 | 0 | -1 | 1 | 0 | 70 | 69.02 |
| 29 | 0 | 0 | 0 | 0 | 85 | 87.98 |

Table 1 Box Behnken design based experimental conditions for the decolourization of MG dye by composite *Mucor sp*
