# Supplementary material for: Preparation and characterization of low-cost adsorbents for the efficient removal of malachite green using response surface modeling and reusability studies
Source: Sci Rep. 2023 Mar 18;13:4493. doi: 10.1038/s41598-023-31391-4 (PMC10024755; doi:10.1038/s41598-023-31391-4)
Supplement: Supplementary file 6 — Supplementary Table 2. [file 41598_2023_31391_MOESM6_ESM.docx]

| Source | Sum of Squares | df | Mean Square | F-value | p-value |  |
| --- | --- | --- | --- | --- | --- | --- |
| Model | 3067.39 | 14 | 219.10 | 60.99 | < 0.0001 | significant |
| A-ph | 52.50 | 1 | 52.50 | 14.61 | 0.0019 |  |
| B-Tp | 0.2821 | 1 | 0.2821 | 0.0785 | 0.7834 |  |
| C-Con | 0.0030 | 1 | 0.0030 | 0.0008 | 0.9773 |  |
| D-Dose | 28.43 | 1 | 28.43 | 7.91 | 0.0138 |  |
| AB | 25.00 | 1 | 25.00 | 6.96 | 0.0195 |  |
| AC | 210.25 | 1 | 210.25 | 58.52 | < 0.0001 |  |
| AD | 9.92 | 1 | 9.92 | 2.76 | 0.1188 |  |
| BC | 85.01 | 1 | 85.01 | 23.66 | 0.0003 |  |
| BD | 211.12 | 1 | 211.12 | 58.76 | < 0.0001 |  |
| CD | 0.0342 | 1 | 0.0342 | 0.0095 | 0.9236 |  |
| A² | 2210.71 | 1 | 2210.71 | 615.34 | < 0.0001 |  |
| B² | 566.31 | 1 | 566.31 | 157.63 | < 0.0001 |  |
| C² | 171.70 | 1 | 171.70 | 47.79 | < 0.0001 |  |
| D² | 122.32 | 1 | 122.32 | 34.05 | < 0.0001 |  |
| Residual | 50.30 | 14 | 3.59 |  |  |  |
| Lack of Fit | 31.85 | 10 | 3.18 | 0.6906 | 0.7115 | not significant |
| Pure Error | 18.45 | 4 | 4.61 |  |  |  |
| Cor Total | 3117.69 | 28 |  |  |  |  |
| Std. Dev. | 1.90 |  | | | **R²** | 0.9839 |
| Mean | 72.55 |  | | | **Adjusted R²** | 0.9677 |
| C.V. % | 2.61 |  | | | **Predicted R²** | 0.9319 |
|  | | | | | **Adeq Precision** | 24.1583 |

Table 2 Analysis of variance (ANOVA), results for decolourization of MG by immobilized *Mucor sp*.
